# A D-Alanine auxotrophic live vaccine is effective against lethal infection caused by *Staphylococcus aureus*

**DOI:** 10.1080/21505594.2017.1417723

**Published:** 2018-02-27

**Authors:** Miriam Moscoso, Patricia García, Maria P. Cabral, Carlos Rumbo, Germán Bou

**Affiliations:** a Department of Microbiology, University Hospital A Coruña (CHUAC) – Biomedical Research Institute A Coruña (INIBIC), A Coruña, Spain; b International Research Center in Critical Raw Materials-ICCRAM, University of Burgos, Burgos, Spain; c Advanced Materials, Nuclear Technology and Applied Bio/Nanotechnology. Consolidated Research Unit UIC-154. Castilla y León. Spain. University of Burgos. Hospital del Rey s/n, Burgos, Spain

**Keywords:** *staphylococcus aureus*, live vaccines, D-alanine auxotrophy, alanine racemase

## Abstract

*Staphylococcus aureus* infections are becoming a major global health issue due to the rapid emergence of multidrug-resistant strains. Therefore, there is an urgent need to develop an effective vaccine to prevent and control these infections. In order to develop a universal immunization strategy, we constructed a mutant derivative of *S. aureus* 132 which lacks the genes involved in D-alanine biosynthesis, a structural component of cell wall peptidoglycan. This unmarked deletion mutant requires the exogenous addition of D-alanine for *in vitro* growth. The aim of this study was to examine the ability of this D-alanine auxotroph to induce protective immunity against staphylococcal infection. Our findings demonstrate that this deletion mutant is highly attenuated, elicits a protective immune response in mice and generates cross-reactive antibodies. Moreover, the D-alanine auxotroph was completely eliminated from the blood of mice after its intravenous or intraperitoneal injection. We determined that the protective effect was dependent on antibody production since the adoptive transfer of immune serum into naïve mice resulted in effective protection against *S. aureus* bacteremia. In addition, splenocytes from mice immunized with the D-alanine auxotroph vaccine showed specific production of IL-17A after *ex vivo* stimulation. We conclude that this D-alanine auxotroph protects mice efficiently against virulent staphylococcal strains through the combined action of antibodies and IL-17A, and therefore constitutes a promising vaccine candidate against staphylococcal disease, for which no licensed vaccine is available yet.

## Introduction

*Staphylococcus aureus* is a versatile Gram-positive bacterium that frequently colonizes the skin and the anterior nares of the nose, being *S. aureus* nasal carriage a well-known risk factor for acquiring an infection [1]. *S. aureus* is a major human pathogen capable of causing a wide range of diseases from skin and soft-tissue infections to more life-threatening diseases such as pneumonia, bacteremia, osteomyelitis, endocarditis and septic shock, among others [2]. In addition, *S. aureus* is also an important pathogen in livestock, being the most frequent cause of bovine mastitis, which is a major problem in the dairy industry [3].

*S. aureus* represents a serious public health problem since both nosocomial and community-associated infections caused by this pathogen have increased in the last two decades. The widespread use of antibiotics has led to an alarming rise in drug-resistant strains, such as methicillin-resistant *S. aureus* (MRSA) and, more recently, the development of vancomycin-resistant staphylococcal strains [4,5]. Vaccination therefore represents an alternative cost-efficient measure for controlling *S. aureus* infections [6]. Several vaccine candidates based on individual cell surface components, such as the polysaccharide capsule or cell wall associated proteins, have been developed and tested in preclinical animal models. Only two of these vaccine candidates, StaphVAX (a polysaccharide conjugate vaccine) and V710 (a vaccine that targets the iron surface determinant B), have reached late-stage clinical phase, but have failed to demonstrate efficacy in Phase III trials [7]. A 4-antigen *S. aureus* vaccine (SA4Ag) is currently under evaluation in a Phase IIB efficacy trial in patients undergoing elective spinal fusion (trial ID: NCT02388165). Due to the complexity of *S. aureus*, a multi-antigen approach that targets cell surface components and secreted virulence factors, and responds to toxins, has been proposed for the successful development of an *S. aureus* vaccine [7,8]. In this context, a live bacterial vaccine has the potential to fulfill these requirements, because it is more likely to mimic natural infection. However, it is not always easy to strike a balance between the attenuation level of a live vaccine and its immunogenic potential. Besides this, live vaccines usually present the risk of reversion to virulent state or of spreading undesired genes, such as antibiotic resistance genes [9]. For several virulent strains, the use of auxotrophic mutants carrying mutations in key metabolic pathways have also been explored for generating potential live vaccine candidates [10-20]. The growth rate of the auxotrophic mutant depends on the availability of the essential metabolite in mammalian tissues. However, the level of attenuation and protective immunity conferred by auxotroph vaccines varies depending on the animal model used and the enzyme disrupted.

Bacterial peptidoglycan is a polymer comprised of repeating disaccharide units of *N*-acetyl glucosamine and *N*-acetylmuramic acid linked by β-1→4 bonds and cross-linked by flexible peptide bridges that vary from one organism to the other. It is found on the exterior of the cytoplasmic membrane in almost all bacteria and it is essential for cell viability. Peptidoglycan contains D-isomers of some amino acids in the stem peptides, particularly D-alanine and D-glutamate, which contribute to the architecture of the murein and provide resistance to most known proteases [21, 22]. In contrast, only trace amounts of D-alanine are found in vertebrates [23]. In *S. aureus*, the stem is the pentapeptide, L-alanine-D-iso-glutamine-L-lysine-D-alanine-D-alanine. The ε-amino group of L-lysine residues of one chain and the carboxyl group of D-alanine in the position four of a neighboring stem are crosslinked by a pentaglycine bridge to provide a high degree of murein cross-linking [24, 25]. Moreover, D-alanine esters in the teichoic acids of many Gram-positive bacteria modulate the polyanionic charge and surface properties of the cell surface [26].

**Figure 1.**
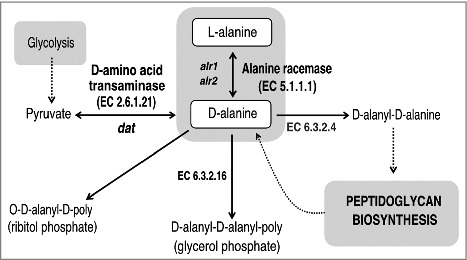
Schematic representation of the D-alanine metabolic pathway in bacteria. The primary route of D-alanine biosynthesis in bacteria is via the reversible interconversion from L-alanine to D-alanine, a reaction catalyzed by alanine racemase (Alr; EC 5.1.1.1). Several Gram-positive bacteria can also favor the synthesis of D-alanine by using the D-amino acid transaminase (Dat; EC 2.6.1.21) to catalyze the interconversion reaction of D-alanine and 2-oxoglutarate to pyruvate and D-glutamate. D-alanine is incorporated into peptidoglycan as a D-alanyl–D-alanine dipeptide, where it is involved in cross-linking of adjacent peptidoglycan strands. In *S. aureus* and many other Gram-positive bacteria, D-alanine esters decorate the teichoic and lipoteichoic acids of the cell wall.

There are two enzymes that catalyze the biosynthesis of D-alanine: alanine racemase and D-amino acid transaminase (Fig. 1). Alanine racemase (Alr, EC 5.1.1.1) is a pyridoxal-5′-phosphate dependent enzyme which catalyzes the reversible racemization of L-alanine and D-alanine [27]. D-amino acid transaminase (Dat, EC 2.6.1.21) catalyzes the transamination reaction between various D-amino acids and α-keto acids. Alanine racemase is a ubiquitous enzyme in bacteria, but is absent in most higher eukaryotes, which has made this enzyme an attractive target for antimicrobial drug development [28]. Bacterial genomes usually contain one or two independent genes coding for two alanine racemase isozymes. The *alr1* gene encodes an anabolic alanine racemase 1 (Alr1), which is expressed constitutively at low level [29]. The *alr2* gene is induced by L-alanine and encodes an alanine racemase 2 (Alr2 or DadX) which is associated with the catabolic function [30]. Several authors have demonstrated that inactivation of both genes encoding two isoforms of alanine racemase, Alr1 and Alr2 (or DadX) is lethal to those bacteria that are dependent on these enzymes for D-alanine biosynthesis, such as *Salmonella typhimurium* or *Escherichia coli* [29, 30]. However, in bacteria with an alternate pathway to convert L-alanine into D-alanine using Dat, such as *Listeria monocytogenes*, it is necessary to inactivate both *alr* and *dat* genes in order to obtain D-alanine auxotrophy [31].

Genes that encode alanine racemase have been used as non-antibiotic-based selectable markers in several bacterial species [32-35], and their potential applications for DNA vaccines have been reported elsewhere [36, 37]. An alanine racemase deficient mutant of *L. monocytogenes* has demonstrated the ability to induce a protective immune response in mice, but only when it was inoculated together with D-alanine, thus limiting its use as a vaccine against listeriosis or as a vaccine vector against other bacterial pathogens or viruses [31, 38]. The potential of the *alr* mutants of lactic acid bacteria as antigen delivery systems, using the tetanus toxin fragment C as model antigen, has also been evaluated in mice [39].

There are few previous studies regarding alanine racemase in *S. aureus*. The gene cluster containing the *alr* gene was identified in *S. aureus* BB255 and the interruption of this gene cluster by insertional mutagenesis lead to a drastic loss of D-alanyl substituents of the lipoteichoic acids and to an altered autolytic behavior [40]. The crystal structure of *S. aureus* alanine racemase from the Mu50 strain has been elucidated [28].

Recently, we reported a strategic platform for the design of whole-cell bacterial vaccines which are auxotrophic for D-glutamate [41]. In this study, we describe the construction and characterization of an unmarked triple deletion mutant of *S. aureus* 132 that results in D-alanine auxotrophy. We will evaluate this mutant's potential as an *S. aureus* live vaccine candidate by investigating its level of attenuation, growth *in vivo* and efficacy at conferring protective immunity in mice.

## Results

### *Construction and characterization of* S. aureus *alanine racemase deficient mutants*

Analysis of the genome sequence of *S. aureus* 132 (GenBank accession number ACOT01000000) revealed two putative alanine racemase genes: *alr1* and *alr2* [42]. When we compared the *S. aureus* 132 genome with other *S. aureus* chromosomes thus far sequenced, we found that the *arl1* gene in the contig ACOT01000040 of *S. aureus* 132 differs by only one nucleotide from the coding sequence (NC_002951) of *S. aureus* subsp. *aureus* COL that encodes a 382 amino acid protein. Likewise, the contig ACOT01000028 contains the *alr2* gene that differs by one nucleotide of the coding sequence (NC_007795) of *S. aureus* subsp. *aureus* NCTC 8325 which encodes a 361 amino acid protein. The Alr1 protein encoded by *S. aureus* 132 has 72% amino acid sequence similarity (38% identity) with Alr1 from *Bacillus subtilis* 168 and 63–67% amino acid sequence similarity with the homolog proteins of *L. monocytogenes* serotype 4b, *Streptococcus pneumoniae* serotype 4 and *Mycobacterium tuberculosis*. On the other hand, the protein encoded by *alr2* from *S. aureus* 132 has 61% amino acid sequence similarity (20% identity) with the Alr2 from *B. subtilis* 168. Both alanine racemase from *S. aureus* 132 showed 23.9% amino acid sequence identity (Fig. S1).

**Figure 2.**
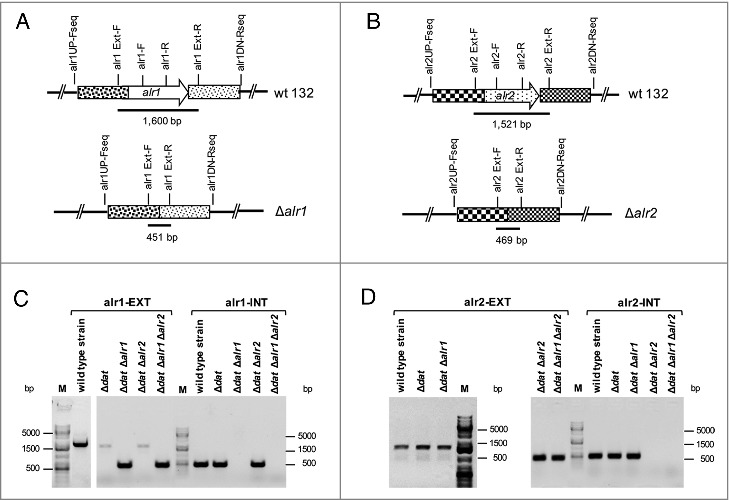
Confirmation by PCR of Δ*alr1* and/or Δ*alr2* gene deletions in *S. aureus* 132 derivatives. A. Map of the *alr1* gene of *S. aureus* 132 and primers used to detect the Δ*alr1* deletion. B. Map of the *alr2* gene of *S. aureus* 132 and primers used to detect the Δ*alr2* deletion. C. Primers alr1EXT-F and alr1EXT-R were used to generate a 1,600 bp fragment (alr1-EXT) from strains carrying the wild type *alr1* allele or a 451 pb fragment from strains carrying the mutant Δ*alr1* allele. Primers alr1-F and alr1-R were used to generate an inner fragment of the *alr1* gene (alr1-INT) of 491 bp only from strains carrying the wild type *alr1* allele. D. Primer alr2EXT-F and alr2EXT-R were used to generate a 1,521 bp fragment (alr2-EXT) from strains carrying the wild type *alr2* allele or a 469 bp fragment from strains carrying the mutant Δ*alr2* allele. Primers alr2-F and alr2-R were used to amplify an inner fragment of the *alr2* gene (alr2-INT) with 519 bp only from strains carrying the wild type *alr2* allele. M, DNA ladder in bp.

Due to the presence of the Dat in *S. aureus*, both *alr* genes were individually deleted from the chromosome of *S. aureus* 132 Δ*dat* mutant strain (Table S1) [41] in order to obtain auxotrophs for D-alanine (Figs. 1, 2 and S2A). Both alanine racemase genes were inactivated by an allelic exchange reaction using pMAD vector as previously described [41, 43], The 132 Δ*dat* mutant strain and the resulting 132 Δ*dat* Δ*alr2* derivative grew normally in TSB without D-alanine, but the double 132 Δ*dat* Δ*alr1* and triple 132 Δ*dat* Δ*alr1* Δ*alr2* mutants required exogenous D-alanine for growth (Fig. S2B). Our results show that the *alr1* gene is necessary for normal growth of *S. aureus* 132 on TSB agar (TSA) without added D-alanine. The *alr1* and/or *alr2* gene deletions in each mutant strain were confirmed by PCR using primers located upstream and downstream of the appropriate gene, plus a set of internal primers located within the sequence. Additionally, the absence of *alr1* and/or *alr2* mRNA in the double or triple mutant strains was confirmed by real time RT-PCR (Table S2 and Figs. 2 and S2C).

The minimal concentration of D-alanine required for growth of the triple mutant of *S. aureus* 132 was determined by plating log-phase cultures on TSA supplemented with different concentrations of D-alanine (from 0.005 to 10 mM). As shown in Fig. S3, the triple mutant showed no visible growth on TSA supplemented with 0.05 mM D-alanine and only a few scattered colonies were visible on a solid medium containing 0.1 mM D-alanine. Above 0.5 mM D-alanine, a confluent growth of the mutant strain was observed.

**Figure 3.**
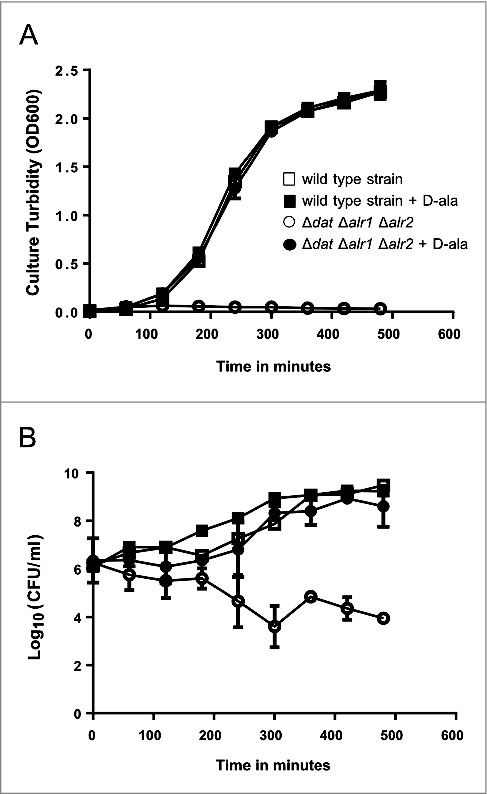
Growth and survival kinetics from *S. aureus* 132 Δ*dat* Δ*alr1* Δ*alr2*. Cells grown in overnight cultures in TSB supplemented with D-alanine were pelleted, washed, and resuspended in fresh medium in the presence or absence of 5 mM D-alanine. A. Growth was monitored by measuring the turbidity (OD at 600 nm) at different times. B. Culture viability (Log_10_ CFU per milliliter) on TSA containing 5 mM D-alanine after growth with or without D-alanine. *S. aureus* 132 Δ*dat* Δ*alr1* Δ*alr2* shows normal growth in TSB supplemented with 5 mM D-alanine, but it is not able to grow without the exogenous supply of this compound. In contrast, the isogenic parental strain grows as per normal in TSB either with or without D-alanine. Squares and circles represent the wild type strain and the triple mutant, respectively in the presence (closed symbols) or the absence (open symbols) of D-alanine.

The growth of *S. aureus* 132 Δ*dat* Δ*alr1* Δ*alr2* in TSB with 5 mM D-alanine was comparable to the growth of the isogenic parental strain in TSB with or without D-alanine with a doubling time of about 35 min. However, no visible growth of the triple mutant in liquid medium lacking D-alanine was observed (Fig. 3A). Likewise, a 2-log-unit decrease in viable cell counts after 8 hours of incubation in TSB was detected for the triple mutant as a result of the limitation of D-alanine in the culture medium (Fig. 3B). Therefore, the *S. aureus* 132 Δ*dat* Δ*alr1* Δ*alr2* strain strictly required an exogenous addition of D-alanine to the culture medium for growth.

**Figure 4.**
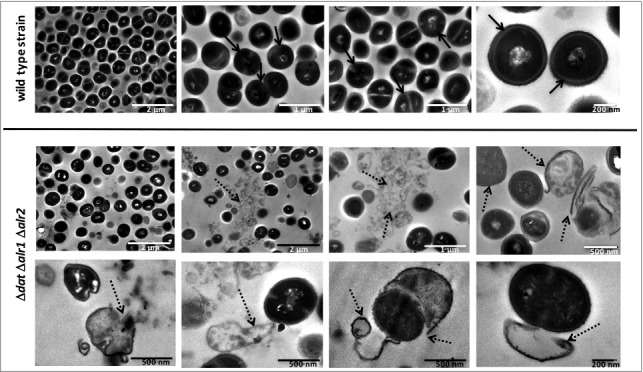
Progressive degeneration of the cell wall and lysis of *S. aureus* 132 Δ*dat* Δ*alr1* Δ*alr2* in the absence of D-alanine. Images were taken with a transmission electron microscope at different magnifications. As control, micrographs of the wild type strain are shown. Black arrows indicate bacteria with an intact cell envelope; dashed arrows indicate cell disruption with loss of intracellular material, projections of the cell wall and release of intracellular content.

The morphological changes of *S. aureus* 132 Δ*dat* Δ*alr1* Δ*alr2* compared to the wild type strain in the presence of different concentrations of D-alanine (from 0.01 to 10 mM) were examined by scanning electron microscopy (SEM). As shown in Fig. S4, the mutant strain displayed a very low cellular density in D-alanine concentrations under 0.1 mM and the grape-like cell clusters typical of *S. aureus* were lost, implying an altered pattern of cell division, which resulted in collapsed cells with irregular surfaces and cell debris. However, with concentrations above 1 mM D-alanine, no differences in cell morphology and density were observed between the wild type and the mutant strain. Micrographs acquired by transmission electron microscopy (TEM) in the absence of D-alanine showed characteristic coccoidal cells of abnormal size, with clear disruption of the cell wall and extrusion of cytoplasmic contents from the mutant cells (Fig. 4).

To estimate the stability of the auxotrophic phenotype of *S. aureus* 132 Δ*dat* Δ*alr1* Δ*alr2* strain, cultures were grown in TSB with 5 mM D-alanine for 11 days. Samples were removed at different days and plated on TSA either with or without D-alanine. In all the resulting plates, no CFU were detected on TSA lacking D-alanine, whereas 7.1 × 10^7^ CFU/mL were counted on TSA supplemented with D-alanine at day 11 of incubation (Fig. S5). Therefore, the reversion frequency was estimated to be lower than 10^−7^ events per cell and per generation.

In order to assess the bacterial response to non-controlled laboratory conditions (i.e. environmental-like conditions), we performed a desiccation tolerance assay. Cultures at log-growth phase of 132 Δ*dat* Δ*alr1* Δ*alr2* and wild type strain were washed and resuspended in TSB, and spotted on sterile membrane filters. After several days, the filters were placed onto TSA plates containing 5 mM D-alanine and incubated at 37°C, and the viability of cells after desiccation was assessed. No difference between the mutant and wild type strains was observed on filters that were not exposed to desiccation (day 0). In contrast, the viability of the triple mutant dramatically decreased from day 32 (Fig. S6) following desiccation.

### *The D-alanine auxotroph of* S. aureus *is attenuated and safe in BALB/c mice*

**Figure 5.**
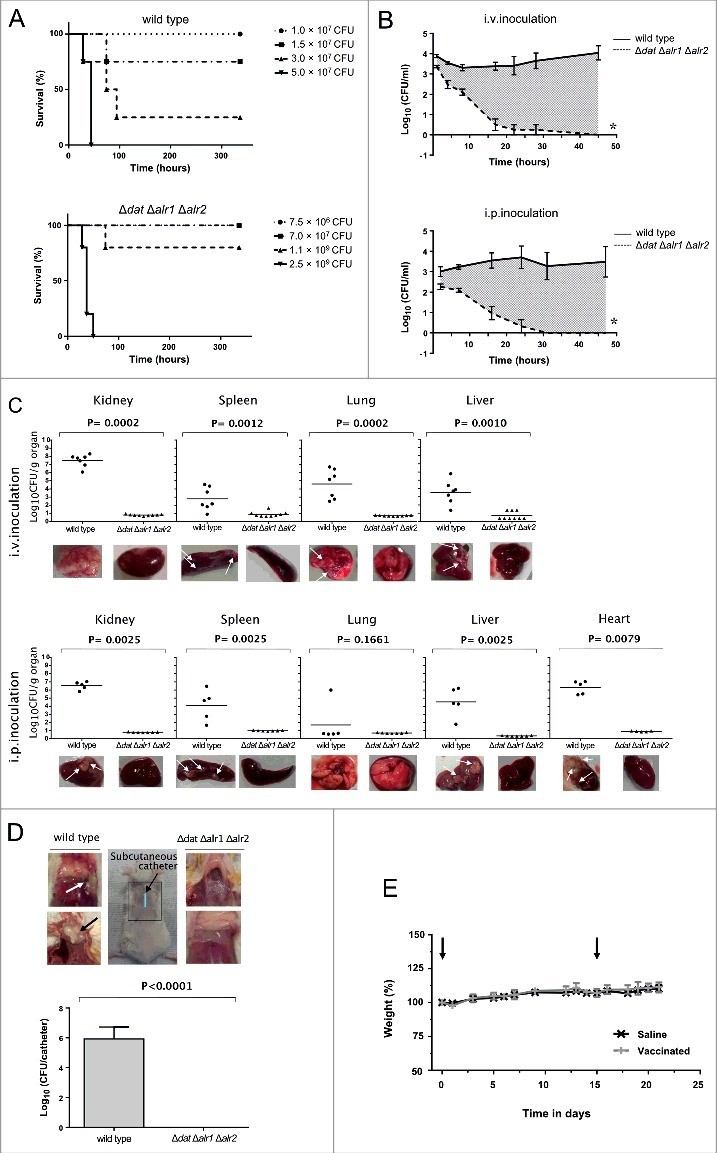
D-alanine auxotrophic strain of *S. aureus* 132 is attenuated for virulence in mice. A. Survival of BALB/c mice (*n* = 4/group) inoculated via i.p. route with *S. aureus* 132 wild type or 132 Δ*dat* Δ*alr1* Δ*alr2* strain at different bacterial doses (CFU), as indicated in the legend. B. Blood clearance of D-alanine auxotroph vaccine candidate. Log_10_ CFU per milliliter of *S. aureus* 132 (on TSA plates) and 132 Δ*dat* Δ*alr1* Δ*alr2* (on TSA containing 5 mM D-alanine) recovered over time from blood of BALB/c mice (*n* = 4/group) after i.v. and i.p. injection with approximately 2.5 × 10^7^ CFU of these strains. Data are mean ± s.e.m. *P < 0.001, according the Student's t test. C. Bacterial dissemination and abscess formation into the different organs after i.v. or i.p. administration of *S. aureus* 132 wild type or 132 Δ*dat* Δ*alr1* Δ*alr2* strains (6 × 10^6^ CFU and 1.9 × 10^7^ CFU, respectively for i.v. inoculation and 2 × 10^7^ CFU and 4 × 10^7^ CFU, respectively for i.p. inoculation) in BALB/c mice (*n* = 5–9/group). At 28 days post-infection, bacterial loads in kidney, spleen, lung, liver and heart (only i.p.) were determined, by plating serial dilution on TSA (parental strain) or TSA plates supplemented with 5 mM D-alanine, as Log_10_ CFU per gram of organ. Each symbol represents an individual mouse, with the horizontal lines showing the mean for each group. *P*-value, according to log-rank (Mantel-Cox) test is indicated in the figure. Representative images of those organs in each group are shown below the graphics. Arrows indicate some bacterial aggregates and abscess formation into the organs. D. Recovery of *S. aureus* 132 wild type (on TSA plates) and the triple mutant Δ*dat* Δ*alr1* Δ*alr2* (on TSA supplemented with 5 mM D-alanine) strains in the catheter infection model in mouse. The implanted subcutaneous catheter segments in BABL/c mice (*n* = 4/group) were inoculated with approximately 1.6 × 10^7^ CFU and bacteria adherent to the catheter were quantified one week after infection. Bars represent the mean of Log_10_ CFU per catheter and the line represent the standard deviation. *P*-value according the Student's t test is indicated. Representative images of the insertion site (square) and the tissue surrounding the catheter with (arrows) or without infection are shown. E. Percent of body weight change in BALB/c mice (*n* = 8/group) after two i.p. injections (days 0 and 14, indicated with arrows) of the vaccine candidate *S. aureus* 132 Δ*dat* Δ*alr1* Δ*alr2* strain (approximately 2 × 10^7^ CFU) compared with mice administered saline.

To assess the impact of D-alanine auxotrophy on *S. aureus* virulence and pathogenesis, we evaluated the ability of 132 Δ*dat* Δ*alr1* Δ*alr2* mutant to cause a sepsis-like infection in mice. Following administration of different doses of bacteria via intraperitoneal (i.p.) injection, the survival rates of BALB/c mice were determined (Fig. 5A). The minimal lethal dose for 100% of infected mice (LD_100_) of the wild type strain, which caused death between 28 and 44 h after injection, was of about 5 × 10^7^ CFU per mouse. In contrast, all animals inoculated with an equivalent dose of the triple mutant (7 × 10^7^ CFU per mouse) displayed no obvious signs of illness, appeared active and healthy, and survived infection over the 14-day duration of the experiment. Using this acute systemic model, a very high dose of bacteria (2.5 × 10^9^ CFU) was required to cause death to all mice inoculated with the mutant strain between 28 and 50 hours after injection. Thus, the simultaneous deletion of *alr1, alr2* and *dat* genes in *S. aureus* 132 resulted in significant virulence attenuation in this mouse model.

To assess the safety of administration of the D-alanine auxotroph mutant strain as a vaccine, the number of bacteria in the blood of infected BALB/c mice was examined at various times after infection with 2.5 × 10^7^ CFU of *S. aureus* 132 and 132 Δ*dat* Δ*alr1* Δ*alr2* strains, by intravenous (i.v.) or i.p. routes. As indicated in Fig. 5B, the wild type strain multiplied in the blood and maintained a high level of bacteremia during the course of the experiment. In contrast, the D-alanine auxotroph of *S. aureus* failed to replicate significantly and was cleared from blood regardless of the administration route.

To determine whether the D-alanine auxotrophic strain was unable to spread to the internal organs and survive via abscess formation, BALB/c mice (*n* = 5–9/group) were injected i.v. or i.p. with *S. aureus* 132 (6 × 10^6^ CFU or 2 × 10^7^ CFU for i.v. or i.p. administration, respectively) or 132 Δ*dat* Δ*alr1* Δ*alr2* strain (i.v., 1.9 × 10^7^ CFU or i.p., 4 × 10^7^ CFU). Four weeks after infection, animals were subjected to gross necropsy and the organs (kidney, spleen, lung, liver and heart) were removed to determine bacterial loads. As indicated in Fig. 5C, infection with the wild-type strain resulted in high tissue bacterial loads that corresponded in most cases with visible surface abscesses. In contrast, D-alanine auxotrophic strain could no longer be isolated from homogenized tissues, and none abscess formation was detected.

Next, we used a catheter infection model in mouse to mimic a device-associated staphylococcal infection and to further evaluate the *in vivo* safety of the D-alanine auxotrophic strain. The catheter segments were subcutaneously implanted in BABL/c mice (*n* = 4/group) and inoculated with approximately 1.6 × 10^7^ CFU of *S. aureus* 132 wild type or the triple mutant Δ*dat* Δ*alr1* Δ*alr2* strains. One week after infection, no adherent bacteria were recovered from catheters inoculated with the D-alanine auxotrophic strain that showed no lesions in the surrounding tissue, in contrast with catheters inoculated the wild type strain, which were infected and purulent (Fig. 5D).

In addition, we observed that BALB/c mice (*n* = 8/group) administered with two injections of the D-alanine auxotrophic strain (approximately 2 × 10^7^ CFU) via i.p. route in a 14-day period did not show differences in the body weight compared to mice administered saline (Fig. 5E).

### *D-alanine auxotrophic strain of* S. aureus *132 confers protection against infection with the parental strain*

**Figure 6.**
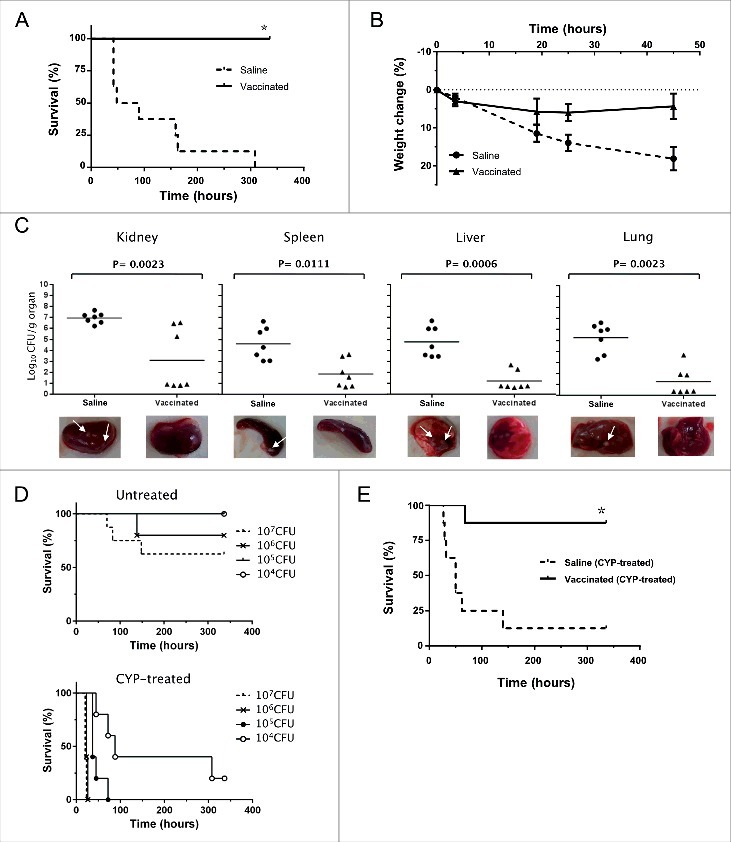
Vaccinated mice are protected against infection with *S. aureus* 132. A. Survival of BALB/c mice (*n* = 8/group) immunized via i.p. with *S. aureus* 132 Δ*dat* Δ*alr1* Δ*alr2* (two dose-schedule, approximately 2.5 × 10^7^ CFU) after challenge with a lethal dose of *S. aureus* 132 (1.2 × 10^8^ CFU per mouse) *P < 0.0001 is significantly different from no vaccination control (Log-rank Mantel-Cox test). B. Percentage weight change relative to starting body weight (preinfection) in BALB/c mice (*n* = 6/group) after challenge with a sub-lethal dose of *S. aureus* 132 (2.4 × 10^7^ CFU per mouse) on day 21. Vaccinated and control mice were injected via i.p. with *S. aureus* 132 Δ*dat* Δ*alr1* Δ*alr2* (two dose-schedule, approximately 3 × 10^7^ CFU per mouse) or saline, respectively. C. Bacterial loads in kidney, spleen, liver and lung obtained from vaccinated and control mice 5 days after infection with *S. aureus* 132. BALB/c mice (*n* = 7/group) were immunized via i.p. with *S. aureus* 132 Δ*dat* Δ*alr1* Δ*alr2* (two dose-schedule, approximately 3 × 10^7^ CFU per mouse) or saline, and challenged with a sub-lethal dose of *S. aureus* 132 (2 × 10^7^ CFU per mouse). Each symbol represents an individual mouse, with the horizontal lines showing the mean for each group. *P*-value, according to log-rank (Mantel-Cox) test is significantly different from no vaccination control in all four organs. Representative images of those organs in each group are shown below the graphics and arrows indicate some bacterial aggregates and small abscess formation into the organs of saline group. D. *S. aureus* 132 infection in leukopenic mice. BALB/c mice (*n* = 5/group) untreated or treated with cyclophosphamide (CYP, three doses of 100 mg/kg via i.p. route) in 48-h intervals were inoculated via i.p. route with *S. aureus* 132 wild type strain at different bacterial doses (CFU), as indicated in the legend, and survival was recorded over 14 days post-infection. E. Vaccine protection of leukopenic mice against *S. aureus* 132 infection. Vaccinated and control BALB/c mice (*n* = 8/group) were injected via i.p. with *S. aureus* 132 Δ*dat* Δ*alr1* Δ*alr2* (two dose-schedule, approximately 3 × 10^7^ CFU per mouse) or saline, respectively. Animals were treated with CYP (three doses of 100 mg/kg via i.p. route) in 48-h intervals, one week before challenge by i.p. injection with *S. aureus* 132 with 7.5 × 10^4^ CFU on day 21 following the first immunization. Survival of mice was recorded over 14 days. *P < 0.005 is significantly different from no vaccination control (Log-rank Mantel-Cox test).

Using a mouse model of acute lethal infection, we first investigated the protective efficacy of the vaccine candidate against *S. aureus* 132 infection (Fig. 6). Mice (*n* = 8/group) were immunized via i.p. route with two doses of the D-alanine auxotrophic strain (approximately 2.5 × 10^7^ CFU per mouse) in a 14-day period. Seven days after the booster injection, mice challenged with a lethal dose of the wild type strain via i.p., were monitored for the clinical signs of illness for 14 days, and the mortality rates were noted. Twenty-four hours after bacterial challenge, all animals (vaccinated and non-vaccinated mice) showed weight loss, piloerection and decreased mobility. However, these clinical signs were more evident in the non-vaccinated group. Finally, vaccinated mice recovered their initial body weight within 7–10 days after challenge, were active and appeared healthy, and had a 100% survival rate at the end of the experiment (Fig. 6A). In contrast, the non-vaccinated mice started to die 42 h post-challenge and all mice succumbed to infection during the observational period. Moreover, we observed that non-vaccinated mice showed about 20% weight loss after challenge with a sub-lethal dose of *S. aureus* 132 (Fig. 6B), indicating a severe disease condition.

The protective effect of vaccination was further characterized by measurement of bacterial loads in different tissues (kidney, spleen, liver and lung) obtained from vaccinated and control mice after challenge with *S. aureus* 132 strain. BALB/c mice (*n* = 7) received two i.p. injections of the vaccine strain (approximately 3 × 10^7^ CFU per mouse) at days 0 and 14. Seven days after the second immunization, these mice were challenged with 2 × 10^7^ CFU of *S. aureus* 132. Five days after infection, all mice were euthanized and bacterial counts in homogenates of kidney, spleen, liver and lung were determined. As illustrated in Fig. 6C, there was a marked decrease in the renal, splenic, hepatic and pulmonary *S. aureus* 132 bacterial loads (3 log-unit reduction) in the vaccinated mice when compared to control mice, which were administered saline solution only. This indicates a protective effect of vaccination against bacterial dissemination.

In addition, we used a leukopenic mouse model to analyze if the D-alanine auxotrophic strain may protect immunocompromised mice against *S. aureus* 132 infection. Firstly, to establish the staphylococcal infection in leukopenic mice, the LD_100_ of the wild type strain was determined in BALB/c mice (*n* = 5/group) untreated or treated with cyclophosphamide (CYP, three doses of 100 mg/kg via i.p. route) in 48-h intervals were inoculated with different doses of *S. aureus* 132 via i.p. injection. As indicated in the survival curves of Fig. 6D, all leukopenic mice (CYP-treated) succumbed to the infection with 3 × 10^6^ CFU of *S. aureus* 132 within 26 h after challenge, whereas this dose caused only 20% mortality in immunocompetent mice (untreated). In addition, 100% of leukopenic mice infected with 2 × 10^5^ CFU of *S. aureus* 132 died within 72 h after challenge and, a dose of 1 × 10^4^ CFU of *S. aureus* 132 caused 80% mortality over the 14-day observation period (Fig. 6D).

Next, BALB/c mice (*n* = 8/group) immunized with D-alanine auxotroph vaccine (two-dose schedule, approximately 3 × 10^7^ CFU) were treated with CYP and challenged with 7.5 × 10^4^ CFU of *S. aureus* 132 on day 21. Most CYP-treated animals (non-vaccinated and vaccinated) adopted a hunched posture, accompanied by lethargy and a body weight loss exceeding 10%. However, all leukopenic mice in the saline group died within 5 days after challenge, while only and one out of eight mice sucumbed to infection in the vaccinated group over the 14-day observation period (Fig. 6E). Moreover, one week after challenge, the vaccinated leukopenic mice appeared active and healthy, and started to recover their body weight.

### *D-alanine auxotrophic strain generates antibody titers against* S. aureus

**Figure 7.**
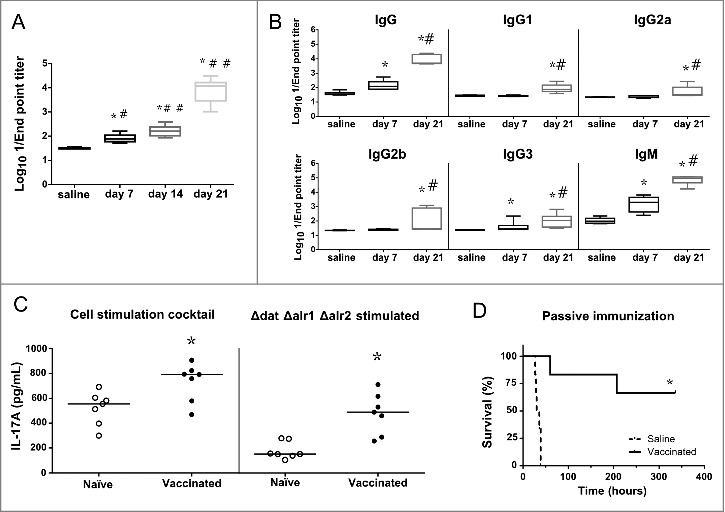
Humoral and cellular immune responses after vaccination. A. Log_10_ 1/Endpoint titer of IgG antibodies produced against the isogenic Δ*spa* strain of *S. aureus* 132 in BALB/c mice (*n* = 6) on post vaccination days 7, 14 (after one immunization) and 21 (two immunizations). BALB/c mice were vaccinated with two doses of *S. aureus* 132 Δ*dat* Δ*alr1* Δ*alr2* (2.5 × 10^7^ CFU). The antibody titers were determined by an indirect ELISA. The boxes represent the first and third quartiles; the horizontal line represents the median; the whiskers represent the interquartile range. *P < 0.001 (Mann-Whitney *U* test), compare with saline group. #*P* < 0.01, ##*P* < 0.001, compared with the preceding condition. B. Log_10_ 1/Endpoint titer of total IgG and subclasses (IgG1, IgG2a, IgG2b, IgG3) and IgM antibodies produced against *S. aureus* 132 Δ*spa* in sera of BALB/c mice vaccinated with two doses of *S. aureus* 132 Δ*dat* Δ*alr1* Δ*alr2* (3 × 10^7^ CFU). Sera was obtained seven days after the first and second immunizations (days 7 and 21, respectively). *P < 0.005 (Mann-Whitney *U* test), compared with saline group. #*P* < 0.05, compared with the day 7. C. Vaccination with *S. aureus* D-alanine auxotrophic strain triggers IL-17A cytokine-secreting T-cells. BALB/c mice (*n =* 7/group) were immunized twice (days 0 and 14) with *S. aureus* 132 Δ*dat* Δ*alr1* Δ*alr2* (5 × 10^7^ CFU per mouse) or administered saline. At day 54 after the second immunization, splenocytes were isolated and ex-vivo restimulated (5 × 10^6^ cells per well) with the vaccine strain (5 × 10^7^ CFU per well) for 48 h. As positive control, mouse splenocytes were cultured with 1X Cell Stimulation Cocktail. The symbols represent the result for each individual animal, and the horizontal line represents the median. There was a significant difference (*P = 0.0023, Mann-Whitney *U* test) in the concentration of IL-17A in splenocyte supernatants of vaccinated mice compared to that of naïve mice. D. Passive antisera transfer from immunized mice protects against *S. aureus* acute lethal infection in naïve mice. BALB/c mice (*n* = 6/group) were administered with antisera (immune or naïve serum) 3.5 h before challenge with *S. aureus* 132 (6 × 10^7^ CFU). Survival was monitored daily for 14 days. *P = 0.0008, according to log-rank (Mantel-Cox) test.

To evaluate the humoral immunity underlying the protection elicited by the D-alanine auxotroph vaccine candidate, we measured antibody titers in sera from immunized mice by ELISA. The *S. aureus* 132 Δ*spa [*42], a protein A-deficient strain, was used as the coating antigen to prevent non-specific interactions of protein A with mouse immunoglobulins. Blood samples from control and immunized mice used in the survival assay (Fig. 6A) were collected post vaccination on days 7, 14 and 21. Fig 7A shows that IgG antibodies were initially detected at day 7 and then slightly increased up to day 14 after a single vaccine administration. A second immunization on day 14, boosted the production of IgG significantly up to day 21.

To evaluate the profile of the humoral response, the serum titers of the total IgG and subclasses, and IgM antibodies were determined by ELISA. The results showed that one immunization (day 7) elicits significant titers of IgG, IgG3 and IgM (Fig. 7B), whereas the second vaccine dose administration significantly boosted these IgG and IgM titers, and stimulated the production of all the IgG subclasses (day 21).

### *Vaccination with D-alanine auxotroph of* S. aureus *suggests a Th17 response in mice*

We evaluated the ability of the D-alanine auxotroph vaccine to stimulate cellular immune responses by measuring the secretion of cytokines (IFNγ, IL-2, IL-4 and IL-17A) in cellular supernatants of splenocytes obtained from vaccinated mice on day 54 after the second immunization and restimulated with vaccine strain. As a positive control, a cell stimulation cocktail was used to induce cytokine production in cell culture. Using a sandwich ELISA, we could not detect a significant increase of IL-4, IL-2 or IFNγ. However, robust IL-17A production was observed after *ex vivo* antigen-specific restimulation with the vaccine strain (Fig. 7C), indicating a profile more polarized towards a Th17 immune response.

### *Passive transfer of antisera from immunized mice provides protection against* S. aureus *acute lethal infection*

In order to examine the protective role of antibodies without the effect of the cellular component of the immune system, we determined whether passive transfer of anti-*S. aureus* sera (anti-Sa) to naïve mice could provide protection against infection with the wild type 132 strain. Pooled immune sera (IgG titer of 1:20,480) were obtained from BALB/c mice immunized with three injections (days 0, 14 and 28) of D-alanine auxotroph vaccine to generate hyperimmune serum and bled 7 days after the last injection. The immune (anti-Sa) or control sera were injected into naïve mice (*n* = 6 per group) 3.5 h before challenge with *S. aureus* 132 (approximately 6.2 × 10^7^ CFU). All mice that were passively immunized with control serum succumbed to infection with *S. aureus* 132 within 45 h after injection (Fig. 7D). In contrast, four out of six mice treated with anti-Sa serum completely resolved the infection during the observation period. These results support the idea that passive transfer of immune serum is protective against *S. aureus* acute lethal infection in mice.

### *The D-alanine auxotroph vaccine generates cross-protective antibodies against* S. aureus *heterologous strains*

**Figure 8.**
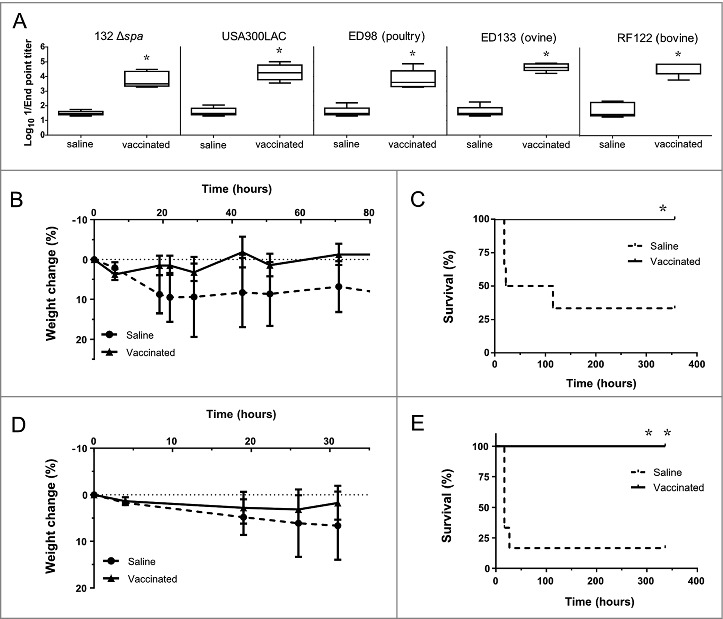
Immunization with D-alanine auxotrophic vaccine elicits cross reactive antibodies and protects against staphylococcal infections. A. Cross reactive IgG antibody titers induced after vaccination against different staphylococcal strains: *S. aureus* 132 Δ*spa*, USA300 LAC, ED98 (from poultry), ED133 (ovine origin) and RF122 (bovine origin). Serum was obtained from BALB/c mice (*n* = 5) two weeks after vaccination with two doses of *S. aureus* 132 Δ*dat* Δ*alr1* Δ*alr2* (about 1.7 × 10^8^ CFU). The boxes represent the first and third quartiles; the horizontal line represents the median; the whiskers represent the interquartile range. *P < 0.05 (Mann-Whitney *U* test,) compared with saline group. B-E. Mice survival (C and E) and percentage of body weight loss (B and D) after vaccination with *S. aureus* 132 Δ*dat* Δ*alr1* Δ*alr2* (two dose-schedule, approximatively 3 × 10^7^ CFU) or saline administration (*n* = 6/group), and challenge with a lethal dose of USA300 LAC (3 × 10^7^ CFU) (B and C) or RF122 (3.5 × 10^7^ CFU) (D and E). *P < 0.05 and **P < 0.005 (Log rank Mantel-Cox test), compared with saline group.

To determine whether the D-alanine auxotroph vaccine generates cross-reactive IgG antibodies against different staphylococcal strains (Table S1), sera from vaccinated mice were obtained at day 28 after a two-dose immunization schedule (days 0 and 14) with the vaccine strain (approximatively 1.7 × 10^8^ CFU). As shown in Fig. 8A, high cross-reactive IgG antibody titers were detected against all tested strains, similar to the IgG titers observed in response to the isogenic Δ*spa* strain of *S. aureus* 132.

To determine the extent of the cross-protection induced by the D-alanine auxotroph vaccine, we challenged vaccinated mice separately with two heterologous *S. aureus* strains: the epidemic clone USA300 [44] and the bovine strain RF122 [45] (Figs. 8B-E). BALB/c mice were immunized with two doses of the vaccine strain in a 14-day period, as previously described. One week after the second dose, all groups (6 mice per group) were challenged with a lethal dose of USA300 LAC (3 × 10^7^ CFU per mouse) or RF122 (3.5 × 10^7^ CFU per mouse), and animals were monitored daily for signs of clinical illness and mortality over a 14-day period. Most non-vaccinated mice exhibited ruffled fur and showed signs of lethargy after infection. Four out of six non-vaccinated mice succumbed to infection with USA300 LAC within 5 days after challenge (Fig. 8C). The infection with the bovine strain RF122 was more acute, and five out of six non-vaccinated mice died within 30 h post-challenge (Fig. 8E). In contrast, all vaccinated mice survived the USA300 LAC and RF122 challenges (Figs. 8C and D), showing only mild clinical signs of illness and a slight body weight reduction, with subsequent recovery (Figs. 8B and D).

## Discussion

The increasing incidence of multidrug-resistant *S. aureus* strains with a rapid dissemination rate, notably MRSA and more recently, strains with reduced susceptibility to vancomycin, is becoming a global issue. Therefore, there is an urgent need to develop vaccines to target this pathogen and to find a universal immunization strategy that protects not only subpopulations at higher risk, such as hemodialysis patients, neonates or cardiac surgery patients, but also the general population, particularly carriers that are at risk for community-associated MRSA infections [46], and where the eradication of carriage would be desirable.

D-alanine is an essential component in the biosynthesis of cell wall peptidoglycan and in the esterification of the teichoic acids of *S. aureus*, and that only trace amounts of this compound are present in human plasma [23]. The presence of two alanine racemase isoforms involved in D-alanine metabolism in Gram-negative bacteria has been described elsewhere [29]. In contrast, most of the Gram-positive bacteria usually possess only one alanine racemase associated with the anabolic function. However, we have identified two putative alanine racemase genes in the genome of *S. aureus* 132, located from the genome sequences of other staphylococcal strains, which also occur in *B. subtilis* [47]. In addition, the presence of the *dat* gene in *Staphylococcus* spp. was also reported [41, 48]. Therefore, in this study we constructed an in-frame alanine racemase- plus D-amino acid transaminase-deficient mutant of *S. aureus* 132 strain for use as a potential vaccine against *S. aureus* infections. Notably, while we have used *Staphylococcus* spp. as a proof of concept, the technology presented here may be easily applicable to other bacteria, similar to those which have recently been reported for D-glutamate auxotrophs [41], Although the unmarked Δ*dat* Δ*alr1* deletion mutant was initially unable to grow on culture medium lacking D-alanine, we have sporadically observed the growth of prototrophic variants after several days of incubation, which indicated the presence of a second alanine racemase activity in this strain, similar to in *B. subtilis* [47]. Therefore, we also eliminated the *alr2* gene, which encodes the potential catabolic alanine racemase. This triple in-frame Δ*dat* Δ*alr1* Δ*alr2* deletion mutant showed an absolute requirement of D-alanine for *in vitro* growth and a stable auxotrophic phenotype.

D-alanine deprivation affected the cell structure of *S. aureus 132* Δ*dat* Δ*alr1* Δ*alr2* and previous reports have also confirmed that mutation of *alr* results in an aberrant cell morphology and cell wall defects, mainly in the septal region of the cell as in *Lactococcus lactis* and *L. plantarum* [49, 50].

The D-alanine auxotroph of *S. aureus* is attenuated, since the LD_100_ of the triple mutant in mice was 50 times higher than that of the parental wild type strain. It is likely that virulence attenuation was due to the blocking of peptidoglycan biosynthesis, since the availability of D-alanine is limited in vertebrate tissues. Moreover, this mutant was rapidly eliminated *in vivo* from blood and the internal organs of mice after i.v. or i.p. administration, suggesting that the use of this D-alanine auxotroph vaccine has no risk for causing a persistent carrier state in the vaccinee nor causing disease. In addition, we showed that there is no risk of adhesion of the auxotrophic strain to artificial devices, like subcutaneous catheters.

Using a mouse model, we also investigated the immunogenic capacity and protective efficacy of the D-alanine auxotroph vaccine candidate against staphylococcal infections. The protection afforded by this D-alanine auxotroph vaccine was evidenced by a reduction in measured bacterial load in organs (kidney, spleen, heart, liver and lung), the absence of abscess formation and enhanced survival of immunized mice following challenge with the parental strain. In addition, we investigated the efficacy of this vaccine in immunocompromised mice, using an infection model in leukopenic mice treated with CYP. Leukopenic mice were highly susceptible to the challenge with *S. aureus* 132, since a reduction by 3 logarithmic units of the LD_100_ was observed. Immunization of mice with the D-alanine auxotrophic strain prior to treatment with CYP, confers to leukopenic mice protection against a low-dose challenge with *S. aureus* 132. However, deeper studies examining the safety profile of this vaccine candidate, for instance using induced leukopenic mice and/or immune deficient mice, should be carried out for warrant the safe prophylactic application of this vaccine in some target populations such those including immunocompromised patients. Previously, Rauch *et al.* have reported that a cocktail of four surface protein antigens (CflA, FnBPB, SdrD and SpA_KKAA_) provided protection against *S. aureus* bloodstream infection in leukopenic mice; however, this protection was limited to a delay in time to death in the immunocompromised mice [51].

The D-alanine auxotrophic *S. aureus* may retain a limited ability to replicate in the host and our results suggest that this whole-cell vaccine contains all the antigenic determinants that are expressed *in vivo* to induce a protective immune response and generate cross-reactive antibodies. Mice immunized with the vaccine strain were also protected from heterologous challenges with the USA300 LAC strain that produces a high level of α-toxin [52], and RF122 strain, isolated from bovine mastitis [45]. However, the development of an intramammary infection model in mouse is needed for testing efficacy against this clinical indication; and future studies in ruminants about the evaluation of the persistence of the D-alanine auxotrophic strain in a bovine mammary epithelial cells will be necessary to support the potential use of this vaccine in veterinary. Several groups have reported D-alanine auxotrophic mutants of *L. monocytogenes *[31], *Burkholderia pseudomallei* [35] and *Mycobacterium tuberculosis* [53] which have been attenuated for *in vitro* growth within murine macrophages or survival in mice, compared to their isogenic parental strains. Conversely, the alanine racemase-deficient mutant of *Burkholderia mallei* was not attenuated for growth or survival [35]. Unexpectedly, a highly attenuated D-alanine auxotroph of *L. monocytogenes* failed to elicit a protective immune response in mice when it was inoculated without D-alanine added to the inoculum administered via i.v. [31]; and thus, vaccination required the dual administration of both the bacteria and the D-amino acid. Later, new strategies to allow transient endogenous synthesis of D-alanine were explored in order to achieve optimal immunogenicity from the D-alanine-deficient *L. monocytogenes* strain, through the conditional expression of a racemase gene using an IPTG-inducible complementation system [54], or a host-induced recombination system [37].

The IgG antibody subclass distribution elicited after vaccination provides insight about the type of immune response generated: a dominance of IgG1 in mice is indicative of a T helper type 2 (Th2) response that promotes humoral immunity, whereas the IgG2a subclass indicates a cellular response with a Th1 profile. Moreover, both types of Th cells induce the secretion of IgM and IgG3 [55]. In this study, we observed a balanced IgG1 and IgG2a response, suggesting a mixed Th1/Th2 profile. However, the protective role of antibodies in immune defense against *S. aureus* infections has previously been questioned [52]. Recent studies suggest that induction of an antibody response alone may be insufficient, and an appropriate vaccine-induced T cell response may be needed to fully protect against *S. aureus* infections [56]. In mice, Th1 cells produce IFNγ, IL-2 and IL-12 cytokines, which are involved in a cellular response, whereas IL-4, IL-5, IL-6, IL-10 and IL-13 are associated with a Th2 response, which mediate B cell activation [57]. In this study, we did not find that the cytokine response in the vaccinated mice was directed towards a clear Th1 or Th2 immune response. Nevertheless, our results demonstrate that immunization with this D-alanine auxotroph vaccine induced a profile more polarized towards a Th17 immune response, considering the increment of IL-17A production by the splenocytes isolated from vaccinated mice. Several authors have shown that IL-17A produced by Th17 cells in response to vaccination contributed to protection against skin and systemic *S. aureus* infections [58-63]. Furthermore, a mouse model of *S. aureus* carriage has confirmed that decolonization is mediated through IL-17A expression and neutrophil influx [64]. Remarkably, Joshi *et al [*65]. have pointed to a critical role of Th17 cells producing IL-17A in IsdB-mediated vaccination against invasive *S. aureus* infection in mice. In addition, in a blinded randomized trial, preoperatively undetectable IL-2 and IL-17A levels were associated with mortality in V710 recipients after postoperative *S. aureus* infections [66].

To our knowledge, this is the first time that a D-alanine auxotroph of *S. aureus* has been tested as an experimental live vaccine against *S. aureus* infections. Although new insights about the protective efficacy and immunogenic potential of this vaccine are presented here, further studies will be necessary to determine the appropriate route for immunization and elucidate the exact contribution of Th17 cells in defense mediated by this D-alanine auxotroph vaccine against staphylococcal infections.

## Materials and methods

### *Construction of alanine racemase deficient mutants of* S. aureus

To generate unmarked and stable in-frame deletions of the *alr1* and/or *alr2* genes of *S. aureus* 132 Δ*dat*, we used an allelic exchange system between the chromosomal genes and the temperature-sensitive shuttle plasmid pMAD as previously described [41, 43]. Detailed descriptions of the construction of *S. aureus* mutants can be found in the Supplemental Material.

### *Growth and viability of D-alanine auxotrophic strain of* S. aureus

*Staphylococcus aureus* 132 and 132 Δ*dat* Δ*alr1* Δ*alr2* strains were grown overnight at 37°C in TSB containing 5 mM D-alanine. Then, bacterial cultures were centrifuged and the pellets were washed with TSB and inoculated at an initial OD_600_ of 0.02 into TSB and TSB supplemented with 5 mM D-alanine. Cultures were incubated at 37°C under agitation and samples were removed every hour up to 8 h for measurement of the culture turbidity (OD_600_) and determination of viable counts (colony-forming units, CFU) on TSA containing 5 mM D-alanine. All experiments were performed in triplicate.

### Electron Microscopy

Samples for electron microscopy were prepared as previously described [41]. Detailed protocols can be found in Supplemental Material.

### Ethics statement

Animal experiments were performed according to the recommendations and the guidelines of the European Union (Directive 2010/63/EU) and current national legislation (RD 53/2013) on the protection of animals used for scientific purposes. The Animal Experimentation Ethics Committee of University Hospital A Coruña (CHUAC) approved all the experiments involving animals in this study (Project ID number: 15002/2013/07). For details of breeding and housing conditions of animals, anesthesia and euthanasia methods, please see Text S1 in the Supplemental Material.

### Mouse immunizations

For active immunizations, *S. aureus* 132 Δ*dat* Δ*alr1* Δ*alr2* strain was grown in TSB supplemented with 5 mM D-alanine at 37ºC shaking at 180–210 rpm until an OD_600_ of 0.7. After centrifugation at 3,900 × g for 20 min, bacteria were washed with 0.9% NaCl (saline) and suspended at the desired concentration in saline solution. Inoculum concentrations were verified by plate counts. BALB/c mice were immunized by i.p. injection with 250 µl of the bacterial suspension following a two-dose schedule (days 0 and 14). Mice in the control group were administered saline solution at the same days. Blood samples (50-100 µL) were collected from the submandibular vein of anesthetized mice at the indicated time points. Sera were separated from the blood clot by centrifugation (2,500 × g, 20 min) and stored at –50ºC for further analysis.

For passive immunization experiments, pools of sera were prepared from BALB/c mice (*n* = 5) administered three i.p. injections of *S. aureus* 132 Δ*dat* Δ*alr1* Δ*alr2* strain (approximately 2 × 10^8^ CFU) at 14-day intervals. Blood samples were collected on day 35 from mice under thiopental anesthesia via puncture of the retro-orbital plexus, and immune serum was separated as above. Control serum was obtained from mice injected with saline solution and processed in the same way. Naïve BALB/c mice aged 9-weeks (*n* = 6/group) were injected via i.p. with immune or control serum (250 µL) 3.5 h prior to the challenge with *S. aureus* 132 strain (approximately 6 × 10^7^ CFU). Survival was monitored daily for 14 days.

### Mouse bacteremia model

To prepare inocula for *S. aureus* infections, bacterial strains were grown in TSB at 37ºC in agitation (180-210 rpm) until an OD_600_ of 0.7. After centrifugation (3,900 × g, 20 min), bacteria were washed and suspended in saline solution containing 3% mucin from porcine stomach in PBS [67] to adjust the required infectious dose per mouse. Bacterial concentrations were verified by plating dilutions of the inoculum onto TSA prior to i.p. administration (250 µl) to BALB/c mice. Clinical signs of disease and mortality were checked twice-daily for a period of 14 days.

To evaluate the protective efficacy of the D-alanine auxotroph vaccine, control and vaccinated BALB/c mice (*n* = 6–8/group) were challenged by i.p. with a lethal dose of *S. aureus* virulent strains on day 21, and monitored daily for 14 days to measure the severity of illness, weight loss and survival.

To determine bacterial dissemination to organs, control and vaccinated BALB/c mice (*n* = 7/group) were challenged with a sublethal dose of *S. aureus* 132 on day 21. Five days after infection, the mice were euthanized and bacterial counts from kidney, spleen, liver and lung were determined. The organs were extracted aseptically, weighed and homogenized in sterile NaCl 0.9% using a Retsch MM200 mixer mill. The homogenates were serially diluted and plated on TSA to determine CFU counts. The number of CFU detected in the organs was standardized per 1 g wet organ weight.

To determine the *in vivo* safety profile of the D-alanine auxotrophic strain, blood clearance and bacterial dissemination to intern organs after i.v. or i.p. administration of *S. aureus* 132 Δ*dat* Δ*alr1* Δ*alr2* strain in BALB/c mice were determined and compared with the administration of *S. aureus* 132 wild type. See Supplemental Material and Methods for additional details.

### Immunocompromised mouse model

Leukopenia was induced in mice by treatment with cyclophosphamide (CYP, Sigma) as previously described [51], with minor modifications. Brief, BALB/c mice (*n* = 8/group) were i.p. injected with three doses of 100 mg CYP per kg body weight in 48-h intervals. The treatment began one week prior to the infection with *S. aureus* 132. To confirm leukopenia, 24 h after the last injection blood samples from the submandibular vein of mouse were collected in collection tubes coated with EDTA and mixed with Türk solution for 5 min before counting. Total white blood cells counts were performed manually for each sample using a Neubauer chamber and microscopic examination.

### Catheter infection model in mouse

The catheter infection model was performed as previously described with some modifications to reduce the severity of procedure [68, 69]. For the catheter insertion, BABL/c mice (*n* = 4/group) were anesthetized by i.p. injection of 25 μL of ketamine (100 mg/kg) and medetomidine (0.5 mg/kg), the hair from the back of the animal was removed using electric hair clippers and the skin was disinfected with povidone-iodine. Then, Introcan Safety catheters (24G, 0.7 × 19 mm) were inserted subcutaneously and inoculated with 150 μL (approximately 1.6 × 10^7^ CFU) of *S. aureus* 132 or 132 Δ*dat* Δ*alr1* Δ*alr2* strains. To close the wound with 3M Steri-strip and to revert the anesthesia with 0.5 mg/kg of atipamezole via i.p. injection. One week after insertion of catheter, mice were euthanized by overdose with sodium thiopental. The catheter segments were aseptically removed, placed in sterile tubes with 1 ml of PBS and sonicated for 15 min in a water bath sonicator, and vortexed at high speed for 1 min. Bacterial counts were determined by plating on TSA or TSA containing 5 mM D-alanine. In addition, the tissue surrounding the catheter was also removed, homogenized and bacteria were quantified.

### Detection of IgG and IgM antibodies by ELISA

The levels of staphylococcus-specific immunoglobulin G (total IgG), as well as IgG subclasses and IgM were quantitatively determined in mouse sera with a whole-bacterial cell ELISA in accordance with the previously described protocol [41]. See the Supplemental Material for additional details.

### Detection of cytokines by ELISA

Interferon-gamma (IFN-γ), interleukin (IL)-2, IL-4 and IL-17A were measured in the cellular supernatant of splenocytes isolated from vaccinated and control mice on day 54 after the second immunization. The cytokine levels were measured with a commercial ELISA kit (Affymetrix, eBioscience) according to the manufacturer's instructions. See the Supplemental Material for additional details.

### Statistical analysis

Statistical evaluation of results was performed using GraphPad Prism software package (version 6.01). Means were compared using Student's *t* test for hypothesis testing to compare individual conditions and corresponding control groups. Survival data were compared using the *Log Rank* (*Mantel-Cox*) test. Comparison of the ELISA results between vaccinated and control groups was statistically analyzed using *Mann-Whitney's* nonparametric, unpaired, two-tailed test. Differences were considered statistically significant when *P* was <0.05.

For additional experimental details, please see the Supplemental Material.

## Supplementary Material

2017VIRULENCE0186R1-s02.pdf
