# Supplementary material for: A D-Alanine auxotrophic live vaccine is effective against lethal infection caused by Staphylococcus aureus
Source: Virulence. 2018 Feb 27;9(1):604–20. doi: 10.1080/21505594.2017.1417723 (PMC5955480; doi:10.1080/21505594.2017.1417723)
Supplement: 2017VIRULENCE0186R1-s02.pdf [file kvir-09-01-1417723-s001.pdf]

TEXT S1. SUPPLEMENTAL MATERIALS AND METHODS

Bacterial strains, plasmids and growth conditions

The bacterial strains and plasmids used are listed in Table S1. *Staphylococcus aureus* strains were cultured in Tryptic Soy Broth No. 2 (TSB from Sigma-Aldrich Co. LLC) or in B2 broth,[1] at 37°C with shaking at 180-210 rpm, unless otherwise stated. *S. aureus* 132 is a biofilm-forming MRSA strain isolated at the Clínica Universitaria de Navarra (Pamplona, Spain) by Vergara-Irigaray and others.[2] *Escherichia coli* strains were grown in Luria-Bertani (LB) media with aeration at 37°C. For plasmid selection, ampicillin and erythromycin were added at a concentration of 100 and 10 µg/mL, respectively. X-Gal (5-bromo-4chloro-3-indolyl-β-D-galactopyranoside) was used at a concentration of 150 µg/mL. D-alanine, when needed, was added to TSB or TSA at a concentration of 5 mM, unless otherwise noted.

DNA manipulation

Genomic DNA from *S. aureus* was isolated after lysis with lysostaphin (0.1 mg/mL), using the Wizard Genomic DNA Purification Kit (Promega Biotech Ibérica, S.L.) according to the manufacturer's protocol. Plasmid DNA from *E. coli* was extracted using the High Pure Plasmid Isolation Kit (Roche Diagnostics, S.L.).

Restrictions enzymes, GoTaq DNA polymerase, TSAP Thermosensitive Alkaline Phosphatase and T4 DNA Ligase were purchased from Promega Biotech Ibérica, S.L. and used as recommended by the supplier. Oligonucleotides and REDAccuTaq LA DNA Polymerase were obtained from Sigma-Aldrich Co. LLC. Primers and probes used are listed in Table S2.

The genome of *S. aureus* 132 was previously sequenced and assembled into 44 large contigs by Vergara-Irigaray and others (GenBank accession number ACOT01000000) [2]. We identified putative alanine racemase genes from *S. aureus* 132 by using the nucleotide sequences of *alr1* and *alr2* (*dadX*) genes from *S. aureus* subsp. *aureus* COL (GenBank accession number NC_002951),[3] and *S. aureus* subsp. *aureus* NCTC 8325 (GenBank accession number NC_007795),[4] in the Clone Manager Suite 9 (Sci-Ed Software) and the

tBLASTx (http://www.ncbi.nlm.hin.gov) programs to probe the assembled contigs of *S. aureus* 132. An analysis of amino acid sequence homology between the putative alanine racemase proteins encoded by *S. aureus* 132 and other known alanine racemases from Gram-positive bacteria was performed using ClustalW.

Electroporation procedure

The recombinant plasmids were introduced by electroporation into *E. coli* DC10β,[5] according to the Gene Pulser Xcell Electroporation System instructions (Bio-Rad Laboratories, Inc.) using the following settings: 200 Ω, 18 kV/cm, 25 µF. The *E. coli* DC10β strain is a DNA cytosine methyltransferase mutant of the high-efficiency *E. coli* strain DH10B, and was used as a universal staphylococcal cloning host.[5] Electrocompetent *S. aureus* cells were prepared as described by Monk *et al.*[5] or according a protocol described for *Staphylococcus carnosus* using SMMP medium for recovery of transformed *S. aureus* cells after electroporation.[6] Plasmid DNA (2.5-6 µg) was added to electrocompetent cells (50 µL) at room temperature. Electroporation was then performed using a Gene Pulser Xcell electroporator (Bio-Rad Laboratories, Inc.) with the following settings: 100 Ω, 21 kV/cm, 25 µF or using the Nucleofector II device (Amaxa Biosystems, Inc.) with a specialized program for bacterial transformation. Immediately after the electric pulse, the cells were incubated in 0.8 mL of TSB supplemented with 0.5 M of sucrose or 1 mL of SMMP at 28°C for 1-2 h, before plating on selective medium.

Construction of alanine racemase deficient mutants of *S. aureus*

To generate unmarked and stable in-frame deletions of the *alr1* and/or *alr2* genes of *S. aureus* 132 Δ*dat*, we used an allelic exchange system between the chromosomal genes and the temperature-sensitive shuttle plasmid pMAD as previously described.[7, 8] First, the contiguous upstream and downstream flanking regions (approximately 1,000 bp) of the *alr1* and *alr2* genes were amplified from the *S. aureus* 132 chromosome by PCR and were cloned into MluI-BglII digested pMAD. The recombinant plasmids, containing the Δ*alr1* or Δ*alr2* mutant alleles, were introduced into *E. coli* DC10β strain as an intermediate host for the

transfer of plasmid DNA into *S. aureus* 132 Δ*dat*. Then, stable *alr* mutants were constructed by two successive crossover events as previously described.[7, 8] Resolved mutants were tested for growth on TSA in the presence or absence of D-alanine to identify auxotrophic strains. The gene deletions were confirmed by PCR using specific primers (see Table S2) and sequence analysis (Macrogen Europe, Amsterdam, The Netherlands).

Real-time RT-PCR

The absence of expression of *alr1* and *alr2* genes of the *S. aureus* deletion mutants was confirmed by RT-PCR using the Universal Probe Library (UPL) TaqMan probes (Roche, Germany) and gene specific primers (see Table S2). Total RNA was extracted from log-phase cultures (OD_{600} = 0.5 to 0.6) using the High Pure RNA Isolation Kit (Hoffman-La Roche Ltd.) according to the manufacturer's protocol. The concentration of RNA was determined by measuring the absorbance at 260 nm (A_{260}) in a NanoDrop ND-1000 spectrophotometer. The purity of the RNA was determined using the A_{260}/A_{280} ratio, where a value close to 2.1 was considered pure. Next, 100 ng of RNA was reverse transcribed into single-stranded cDNA. Real-time PCR amplification was carried out with a LightCycler® 480 RNA Master Hydrolisis Probes kit in a LightCycler 480 instrument (Roche Diagnostics, S.L., Germany) with an initial incubation of 65°C for 3 min, a denaturation step at 95°C for 30 s, followed by 45 cycles of target amplification consisting of 15 s at 95°C and 60°C for 45 s, and a final elongation step of 30 s at 40°C. In all cases, the expression levels of the genes of interest were normalized relative to the transcription levels of the housekeeping gene *gyrB*. All RNA extractions and RT-PCR assays were performed in duplicate in two independent experiments.

Determination of D-alanine requirement for growth of *S. aureus* 132 Δ*dat* Δ*alr1* Δ*alr2*

Overnight cultures from *S. aureus* 132 and the isogenic 132 Δ*dat* Δ*alr1* Δ*alr2* derivative were diluted into TSB supplemented with 10 mM D-alanine and incubated at 37°C under agitation until an optical density at 600 nm (OD_{600}) of 0.25 was achieved. Bacteria from log-phase cultures were collected by centrifugation, washed and resuspended in TSB, and then inoculated onto TSA supplemented with different concentrations of D-alanine (0.005 to 10

mM). Plates were incubated at 37°C for 24 h and growth was observed.

Evaluation of phenotypic stability

Overnight cultures from *S. aureus* 132 Δ*dat* Δ*alr1* Δ*alr2* were diluted at an initial OD_{600} of 0.02 into TSB supplemented with 5 mM D-alanine and incubated at 37°C under agitation for up to 11 days. Aliquots were removed at different days and the cell viability was evaluated by plating cells onto TSA in the presence or absence of 5 mM D-alanine. All experiments were performed in triplicate.

Electron Microscopy

Samples for electron microscopy were prepared as previously described [7]. Briefly, bacteria for transmission electron microscopy (TEM) samples were grown at 37°C in TSA containing 10 mM D-alanine. Discrete colonies were spread on Mueller-Hinton agar using a swab, and incubated overnight at 37°C. Cells were then harvested by centrifugation, washed in 0.2 M sodium cacodylate buffer, pH 7.4 and prefixed in 2.5% (w/v) glutaraldehyde for 4 h at room temperature and fixed with 1% (w/v) osmium acetate in 0.2 M sodium cacodylate buffer, pH 7.4. After washing with cacodylate buffer and dehydration with acetone, cells were embedded in SPURR (Spurr's Epoxy Embedding Medium). Ultrathin sections (70 nm) of these samples were stained with 4% (w/v) uranyl acetate and then with 0.4% (w/v) lead citrate, and examined with a JEOL JEM transmission electron microscope at 80 kV.

For scanning electron microscopy (SEM), bacterial strains were grown overnight at 37°C in TSB containing 10 mM D-alanine. After incubation, bacterial cultures were centrifuged and the pellets were washed twice with 0.9% NaCl and resuspended in TSB. These samples were inoculated at an initial OD_{600} of 0.02 into TSB supplemented with different concentrations of D-alanine (0.01, 0.1, 1 and 10 mM) and cultures were incubated at 37°C under agitation for 3 h. Cells were then harvested by centrifugation, washed twice in 0.1 M PBS, pH 7.4, fixed in 4% paraformaldehyde for 30 min at room temperature and washed again twice in 0.1 M PBS, pH 7.4. Each sample was dehydrated in an ethanol series and then dried to the critical point with CO_2 (Bal-Tec CPD 030). One drop of each sample was placed

onto a slide cover and fixed in aluminium supports for gold coating (Bal-Tec SCD 004 sputter coater). All specimens were examined with a Jeol JSM-6400 electron microscope. Samples were observed under × 2,500, × 5,000, × 10,000 and × 20,000 magnification.

Desiccation tolerance assay

To compare the sensitivity of *S. aureus* 132 and 132 Δ*dat* Δ*alr1* Δ*alr2* strains to desiccation stress, culture samples at log-phase growth (OD_{600} = 0.6) were washed and serially diluted in TSB. Drops of 5 µL were spotted onto sterile 0.45 µm mixed cellulose esters membrane filters and maintained at room temperature. At various time points, the filters were placed onto TSB agar plates containing 5 mM D-alanine and incubated at 37°C for 18 h. The desiccation resistance of the cells was determined by comparing the viability of spots before and after stressful conditions between the wild type and the mutant strain. All cultures were performed in triplicate.

Animals

Animals were bred and maintained under specific pathogen-free conditions in the facility at the Centro Tecnológico de Formación de la Xerencia de Xestión Integrada A Coruña (CTF-XXIAC), Servicio Galego de Saúde, with free access to food and water. Female BALB/c mice at 6 to 8 weeks of age were used for active immunizations and at 9-weeks old for passive immunization experiments. Male BALB/c mice aged 6-weeks were used for estimation of cytokine levels produced in mouse spleen homogenates. All mice were anesthetized with 5% sevoflurane in oxygen. After infection, mice were monitored daily for signs of clinical illness (piloerection, hunched posture, decreased mobility, rolling/twisting movements and weight loss) over a period of 14 days. Mice were euthanized with an overdose of thiopental sodium and cervical dislocation at the end of experiments or when animals exhibited a greater than 20% body weight loss or severe signs of rolling/twisting movements.

Bacterial clearance from blood

To investigate bacterial clearance from the blood of mice i.v. inoculated, an equivalent dose of the parental 132 strain and the isogenic 132 Δ*dat* Δ*alr1* Δ*alr2* derivative were independently prepared in saline as above. BALB/c mice (*n* = 4/group) were then injected with 2-3 × 10^7 CFU (100 μL) of the bacterial suspension i.v. via the lateral tail vein. At different times, blood samples were collected from the submandibular vein of mice and the number of CFU was determined by plating serial dilution on TSA (parental strain) or TSA plates supplemented with 5 mM D-alanine (mutant strain). For i.p. administration, bacteria were suspended in saline solution containing 3% mucin from porcine stomach and BABL/c mice (*n* = 4/group) were injected with 2-3 × 10^7 CFU (250 μL) of the bacterial suspension via i.p. route.

Bacterial dissemination to internal organs

In order to determine bacterial dissemination to internal organs, BALB/c mice (*n* = 7-9/group) were injected with 100 μL of the bacterial suspension i.v. via the lateral tail vein (6 × 10^6 CFU of *S. aureus* 132 or 1.9 × 10^7 CFU of 132 Δ*dat* Δ*alr1* Δ*alr2* derivative). At 28 days post-infection, the mice were euthanized and bacterial loads in different organs (kidney, spleen, lung and liver) were determined. The organs were extracted aseptically, weighed and homogenized in sterile NaCl 0.9% using a Retsch MM200 mixer mill. The homogenates were serially diluted and plated on TSA (parental strain) or TSA plates supplemented with 5 mM D-alanine (mutant strain) to determine CFU counts. The number of CFU detected in the organs was standardized per 1 g wet organ weight. For i.p. administration, bacteria were suspended in saline solution containing 3% mucin from porcine stomach and BABL/c mice (*n* = 5-7/group) were injected with 250 μL of the parental 132 strain (2 × 10^7 CFU) or the isogenic 132 Δ*dat* Δ*alr1* Δ*alr2* derivative (4 × 10^7 CFU) via i.p. route. Similarly, at 28 days post-infection, bacterial loads in kidney, spleen, lung, liver and heart were determined as Log_{10} CFU per gram of organ.

Detection of IgG and IgM antibodies by ELISA

The levels of staphylococcus-specific immunoglobulin G (total IgG), as well as IgG

subclassess (IgG1, IgG2a, IgG2b and IgG3) and IgM were quantitatively determined in mouse sera with a whole-bacterial cell Enzyme-linked Immunosorbent Assay (ELISA) in accordance with the previously described protocol.[7] In this assay, *S. aureus* strains were grown in TSB until an OD_{600} of 1, diluted 1:10 in 100 mM carbonate-bicarbonate buffer, pH 9.6, and bacterial cells were fixed to the bottom of ELISA plates (Greine Bio-one #655061; 96-wells, polystyrene, F-bottom) by overnight incubation at 4ºC. For the detection of specific antibodies against *S. aureus* 132, a protein A-deficient strain (132 Δ*spa*) was used to avoid the nonimmune binding of the Fc region of IgGs to protein A.[2,9] After coating, plates were washed five times with phosphate buffered saline (PBS) and blocked with 5% skim milk in PBS for 2 h at room temperature. For other staphylococcal strains, an additional blocking step with rabbit serum (dilution 1:1,000 in PBS) for 1 h at 37ºC was included to reduce background by blocking non-specific interactions of the primary antibody and protein A. Then, the wells were aspirated and washed five times with PBS containing 0.005% Tween-20 (PBS-T). Two-fold serial dilutions of mouse sera in DMEM supplemented with 10% fetal calf serum (FCS from Gibco) were added to wells, and incubated overnight at 4ºC. The plates were again washed with PBS-T and 100 μL of horseradish peroxidase (HRP)-conjugated anti-mouse secondary antibodies (anti-mouse IgG, IgG1, IgG2a, IgG2b, IgG3 or IgM, HRP conjugates produced in rabbit from Sigma-Aldrich) at a dilution of 1:5,000 in DMEM supplemented with 10% FCS was added to each well. After another hour of incubation at room temperature in the dark, the plates were washed five times with PSB-T and 100 μL of 3,3'5,5'-Tetramethylbenzidine (TMB; Sigma-Aldrich) was added to each well. After 2 min, the colour reaction was stopped by adding 50 μL of 1N H_2SO_4 per well. The absorbance was read at 450 nm with a modular microplate reader (Infinite M200 NanoQuant from Tecan Group Ltd.). The endpoint titers for each antibody were defined as the maximum dilution of serum with an absorbance 0.1 point higher than of the sera blank wells.

Detection of cytokines by ELISA

Interferon-gamma (IFN-γ), interleukin (IL)-2, IL-4 and IL-17A were measured in the cellular supernatant of splenocytes isolated from vaccinated and control mice on day 54 after the

second immunization. The cytokine levels were quantified by ELISA with the Mouse Th1/Th2 ELISA Ready-SET-Go! and the Mouse IL-17A (homodimer) ELISA Ready-SET-Go! (Affymetrix, eBioscience, Ltd.) kits according to the manufacturer's instructions. BALB/c male mice (n = 7/group) were sacrificed under proper anesthesia and mice spleens were removed aseptically, mechanically disrupted and washed in saline by centrifugation at 400 × g for 10 min at room temperature. The cell suspension was enriched for lymphocytes using a Histopaque density gradient (Sigma-Aldrich). The cells were suspended in RPMI-1640 medium containing 10% FCS and dispensed into 24-well plates (Costar 3524, flat bottom with lid, polystyrene, sterile) at a concentration of 5×10^6 splenocytes per well. Splenocytes were then ex-vivo restimulated with the vaccine strain (5×10^7 CFU per well) and incubated at 37ºC, 5% CO_2 for 48 h. As positive control, mouse splenocytes were cultured with 1X Cell Stimulation Cocktail (Affymetrix, eBioscience, Ltd.) at 37ºC, 5% CO_2. This cocktail contains phorbol 12-myristate 13-acetate and ionomycin to induce cytokine production in cell cultures. Supernatants from cell cultures were collected after 48 h, and cytokines were detected by a sandwich ELISA protocol. Briefly, the 96-well ELISA plates (Costar 9018 or Nunc Maxisorp) were coated with the capture antibodies (anti-mouse IFN-γ, IL-2, IL-4 and IL-17A) overnight at 4ºC. The ELISA plates were then washed five times with wash buffer (1× PBS, 0.05% Tween-20, pH 7.4) and incubated with 1× ELISA Diluent (PBS supplemented with 10% FCS) at room temperature for 1 h. Next, 100 μL of the culture supernatants from restimulated splenocytes was added to each well followed by incubation overnight at 4ºC for maximum sensitivity. A relative standard curve was created using a serial dilution of each recombinant mouse cytokine protein. Next day, the wells were aspirated and washed five times with wash buffer. Then, 100 μl of biotin-conjugated anti-mouse cytokine (IFN-γ, IL-2, IL-4 and IL-17A) antibody was added to each well and the plates were incubated at room temperature for 1 h. After washing, 100 μL of avidin-HRP conjugate solution was added and the plates were incubated at room temperature for 30 min. After washing, 100 μL of TMB substrate was added and the plates were incubated at room temperature for 15 min. To stop the color reaction, 50 μL of 1 N H_2SO_4 was added to each well. Absorbance was read at 450 nm and 570 nm. The background absorbance from the

microplate was corrected for by subtracting values of 570 nm (reference) from those of 450 nm. The IFN-γ, IL-2, IL-4 and IL-17A cytokine levels in the culture supernatants were estimated by interpolation from the standard curve and were expressed as pg/mL.

TEXT SI REFERENCES

1. Schenk S, Laddaga RA. Improved method for electroporation of *Staphylococcus aureus*. FEMS Microbiol Lett 1992; 73:133-8.
2. Vergara-Irigaray M, Valle J, Merino N, Latasa C, Garcia B, Ruiz de Los Mozos I, et al. Relevant role of fibronectin-binding proteins in *Staphylococcus aureus* biofilm-associated foreign-body infections. Infect Immun 2009; 77:3978-91.
3. Gill SR, Fouts DE, Archer GL, Mongodin EF, Deboy RT, Ravel J, et al. Insights on evolution of virulence and resistance from the complete genome analysis of an early methicillin-resistant *Staphylococcus aureus* strain and a biofilm-producing methicillin-resistant *Staphylococcus epidermidis* strain. J Bacteriol 2005; 187:2426-38.
4. Gillaspy AF, Worrell V, Orvis J, Roe BA, Dyer DW, Iandolo JJ. The *Staphylococcus aureus* NCT8325 genome. In: Fischetti V, Novick R, Ferretti J, Portnoy D, Rood J, eds. Gram-positive pathogens: ASM Press, 2006.
5. Monk IR, Shah IM, Xu M, Tan MW, Foster TJ. Transforming the untransformable: application of direct transformation to manipulate genetically *Staphylococcus aureus* and *Staphylococcus epidermidis*. MBio 2012; 3.
6. Lofblom J, Kronqvist N, Uhlen M, Stahl S, Wernerus H. Optimization of electroporation-mediated transformation: *Staphylococcus carnosus* as model organism. J Appl Microbiol 2007; 102:736-47.
7. Cabral MP, García P, Beceiro A, Rumbo C, Pérez A, Moscoso M, et al. Design of live attenuated bacterial vaccines based on D-glutamate auxotrophy. Nat Comms 2017. doi:10.1038/NCOMMS15480.
8. Arnaud M, Chastanet A, Debarbouille M. New vector for efficient allelic replacement in naturally nontransformable, low-GC-content, gram-positive bacteria. Appl Environ Microbiol 2004; 70:6887-91.
9. Merino N, Toledo-Arana A, Vergara-Irigaray M, Valle J, Solano C, Calvo E, et al. Protein A-mediated multicellular behavior in *Staphylococcus aureus*. J Bacteriol 2009; 191:832-43.

Table S1. Strains and plasmids used in this study.

Strain or plasmid	Relevant features	Reference
***S. aureus* strains**		
S. aureus 132	MRSA clinical isolate	1
132 Δ*dat*	132 derivative, Δ*dat*	2
132 Δ*dat* Δ*alr1*	132 derivative, Δ*dat* Δ*alr1*	This study
132 Δ*dat* Δ*alr2*	132 derivative, Δ*dat* Δ*alr2*	This study
132 Δ*dat* Δ*alr1* Δ*alr2*	132 derivative, Δ*dat* Δ*alr1* Δ*alr2*	This study
132 Δ*spa*	132 derivative, Δ*spa* protein A-deficient	1
FPR3757 (USA300 LAC)	USA300 epidemic clone	3
RF122	ST151 and CC151 strain from bulk milk	4
ED133 (formerly 1174)	ST133 and CC133 strain from ovine mastitis	5
ED98	ST5 and CC5 strain from broiler chicken (skeletal infection)	6
***E. coli* strains**		
E. coli TG1	*supE thi-1 Δ(lac-proAB) Δ(mcrB-hsdSM)5,* $(r_K^- m_K^-)$*[F' traD36 proAB* $lacI^q$*ZΔM15]*	7
E. coli DC10β	Δ*dcm* in the DH10B background [F- mcrA Δ(*mrr-hsdRMS-mcr*BC) *Φ80dlacZΔM15 ΔlacX74 endA1 recA1 deoR Δ(ara,leu)7697 araD139 galU galK nupG rpsL λ-]*	8
**Plasmids**		
pMAD	A temperature-sensitive *E. coli*/*S. aureus* shuttle vector. Amp^R; Ery^R, $bgaB^+$	9
pMAD- Δ*alr1*	pMAD derivative, source of Δ*alr1*	This study
pMAD- Δ*alr2*	pMAD derivative, source of Δ*alr2*	This study

Table S1 References

1. Vergara-Irigaray M, Valle J, Merino N, Latasa C, Garcia B, Ruiz de Los Mozos I, et al. Relevant role of fibronectin-binding proteins in *Staphylococcus aureus* biofilm-associated foreign-body infections. Infect Immun 2009; 77:3978-91.
2. Cabral MP, García P, Beceiro A, Rumbo C, Pérez A, Moscoso M, et al. Design of live attenuated bacterial vaccines based on D-glutamate auxotrophy. Nat Comms 2017. doi:10.1038/NCOMMS15480.
3. Diep BA, Gill SR, Chang RF, Phan TH, Chen JH, Davidson MG, et al. Complete genome sequence of USA300, an epidemic clone of community-acquired meticillin-resistant *Staphylococcus aureus*. Lancet 2006; 367:731-9.
4. Fitzgerald JR, Monday SR, Foster TJ, Bohach GA, Hartigan PJ, Meaney WJ, et al. Characterization of a putative pathogenicity island from bovine *Staphylococcus aureus* encoding multiple superantigens. J Bacteriol 2001; 183:63-70.
5. Ben Zakour NL, Sturdevant DE, Even S, Guinane CM, Barbey C, Alves PD, et al. Genome-wide analysis of ruminant *Staphylococcus aureus* reveals diversification of the core genome. J Bacteriol 2008; 190:6302-17.
6. Lowder BV, Guinane CM, Ben Zakour NL, Weinert LA, Conway-Morris A, Cartwright RA, et al. Recent human-to-poultry host jump, adaptation, and pandemic spread of *Staphylococcus aureus*. Proc Natl Acad Sci U S A 2009; 106:19545-50.
7. Simon R, Priefer U, Pühler A. A broad host range mobilization system for in vivo genetic engineering: transposon mutagenesis in Gram negative bacteria. Nat Biotechnology 1983; 1:784-91.
8. Monk IR, Shah IM, Xu M, Tan MW, Foster TJ. Transforming the untransformable: application of direct transformation to manipulate genetically *Staphylococcus aureus* and *Staphylococcus epidermidis*. MBio 2012; 3.
9. Arnaud M, Chastanet A, Debarbouille M. New vector for efficient allelic replacement in naturally nontransformable, low-GC-content, gram-positive bacteria. Appl Environ Microbiol 2004; 70:6887-91.

Table S2. Oligonucleotides and probes used in this study.

Primer	Sequence (5'→3')[a]
**alr1UP-F(MluI)**	cccacgcgtCATTACTTAAACGCAACA
**alr1UP-R(NotI)**	cccgcggccgcATTACTTCCTCCTGTAAT
**alr1DN-F(NotI)**	cccgcggccgcTATGGTCAGTGCATATAA
**alr1DN-R(BglII)**	cccagatctCTTCCACGATTAGTTG
**alr1-F**	CGAGTTGCCTTAACGGTTCC
**alr1-R**	ACAGACTCACCCGCTTGTAG
**alr1Ext-F**	GCATTAGGCACAGGCTTAGG
**alr1Ext-R**	CAATCGCATGCTTCACACTC
**alr1UP-Fseq**	TCCAGAATTTCGAGCTATTG
**alr1DN-Rseq**	TCACTTCGTCAGTGTATTTC
**alr2UP-F(MluI)**	cccacgcgtGTCATTGCATACTTAGAA
**alr2UP-R(NotI)**	cccgcggccgcTGTATTACACCTCTTTGT
**arl2b-DN-F (NotI)**	cccgcggccgcAGGAGTACATTTCAAATG
**alr2DN-R(BglII)**	cccagatctCTGCTTCTTCATTTCTAT
**alr2-F**	AGTCCGTGAACATCAAATAC
**alr2-R**	CTATAACCGCAATAATCACC
**alr2Ext-F**	GTCTATGACAAACCAACGCC
**alr2Ext-R**	CCTCAGCTACAAGTTTGACC
**alr2UP-Fseq**	CATCAACATCCTGAATTAAGC
**alr2DN-Rseq**	GATGAAGGTAATTTAGCGTC
**datUP (MluI) F**	cccacgcgtGAAACGTATTCATATGAT
**datUP (NotI) R**	cccgcggccgcATATTATTCCTCCACGC
**datDOWN (NotI) F**	cccgcggccgcAATTCTTTCATCATATTT
**datDOWN (BglII) R**	cccagatctGCGAATCTAAACTCGGTA
**DatF**	TATTCAAGCAACGCGTGGTG
**DatR**	AGTTGACGTGTAATTGGGCC
**datExtF**	GTCATGGGTGACGTGACAAC
**datExtR**	GCACCACCTGCTGAATCAAG
**datseqF**	GCCGGTTGTAACAGAAGATG
**datseqR**	CAATTGCCGGGTCTGCAATC
**gyrB#131-left**	CGGTGGCGGATACAAAGT
**gyrB#131-right**	GCGTTTACAACTGATGAACCA
**alr1#78-left**	CAATTGTTAAAGTGCCAGATCAA
**alr1#78-right**	ACCACCTCTACCGACTGTGG
**alr2#136-left**	GGTAAACGCTACCCGATACG
**alr2#136-right**	TGTACATTGCCATCTACTTCAACA

[a]The restriction enzymes sites are underlined.

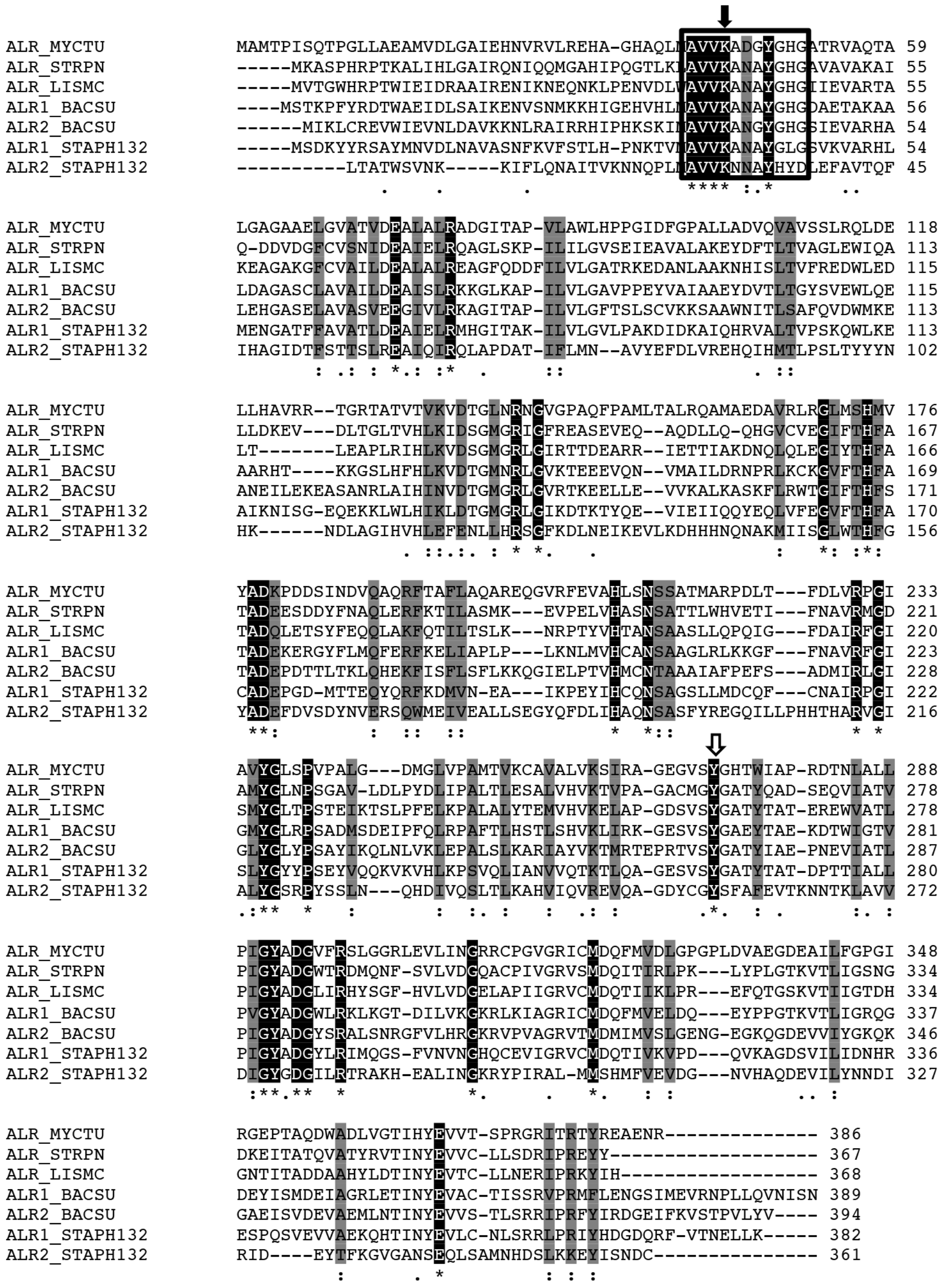

Figure S1. Multiple alignment of amino acid sequences of alanine racemases (Alr) from *S. aureus* 132 (ALR1_STAPH132 and ALR2_STAPH132) and other Gram-positive bacteria. The amino acid sequence alignment of representative Alr was generated with Clustal Omega (1.2.1). Identical residues are shown on a black background and amino acids with similar physicochemical properties are indicated by a gray background. The box encloses the conserved pyridoxal-5'-phosphate (PLP)-binding motif. The black arrow marks the highly conserved PLP-bound lysine, and the white arrow indicates the location of the catalytic tyrosine. The representative Alrs are homologs from *Mycobacterium tuberculosis* ATCC 25618 (ALR_MYCTU), *Streptococcus pneumoniae* serotype 4 (ALR_STRPN), *Listeria monocytogenes* serotype 4b (ALR_LISMC) and *Bacillus subtilis* 168 (ALR1_BACSU and ALR2_BACSU).

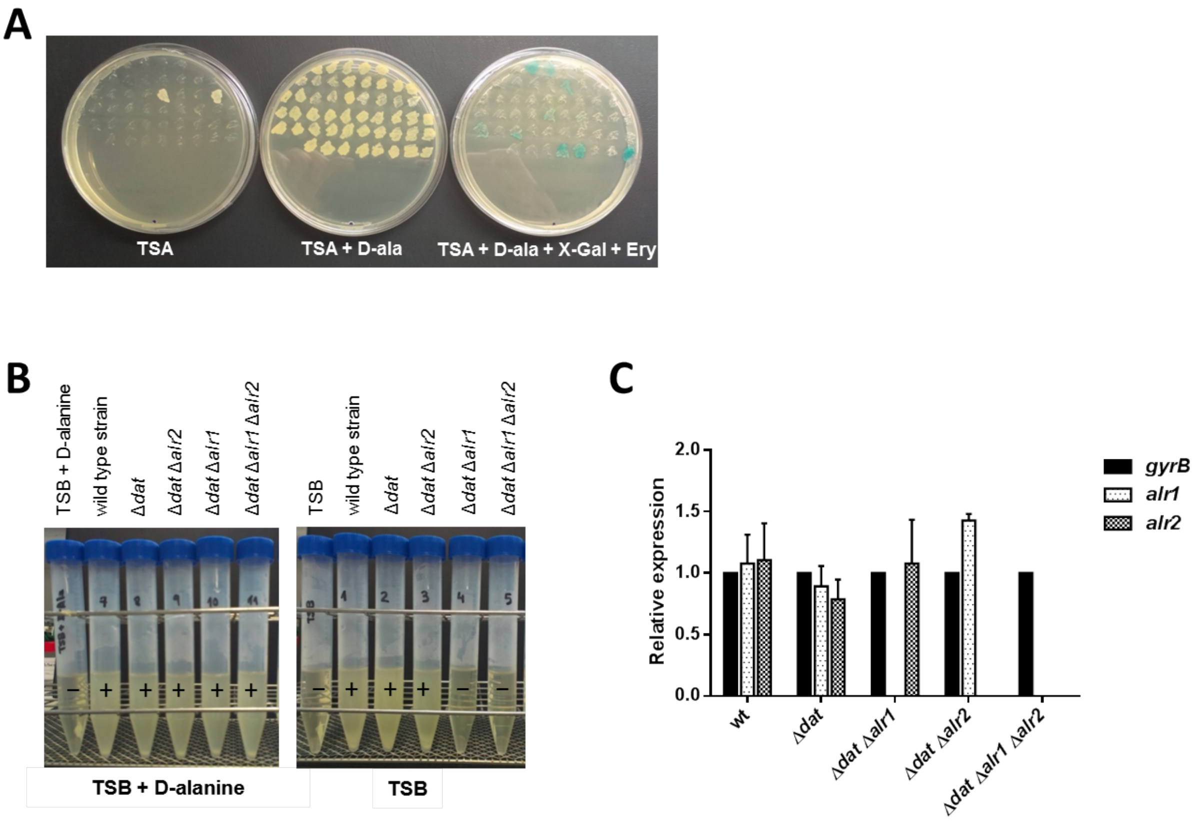

Figure S2. A. Phenotypic screening of D-alanine auxotrophy during the construction of *S. aureus* 132 Δ*dat* Δ*alr1* Δ*alr2* triple mutant. Individual colonies of resolved co-integrants after two successive crossover events were grown on TSA plates supplemented or not with D-alanine (5 mM) and erythromycin (Ery, 10 µg/mL). Resolvants with the Δ*dat* Δ*alr1* Δ*alr2* genotype are erythromycin-sensitive and grew only on TSA with D-alanine. **B.** Growth of the *S. aureus* 132 wild-type strain and the single, double, and triple isogenic mutants (Δ*dat*, Δ*dat* Δ*alr1*, Δ*dat* Δ*alr2* and Δ*dat* Δ*alr1* Δ*alr2*) in TSB supplemented or not with 5 mM D-alanine, after 24 h of incubation at 37°C with agitation. A plus (+) sign indicates growth and a minus (–) sign indicates no growth. *S. aureus* 132 and 132 Δ*dat* Δ*alr2* derivative grow normally in TSB with or without D-alanine. However, 132 Δ*dat* Δ*alr1* and 132 Δ*dat* Δ*alr1* Δ*alr2* derivatives exhibit normal growth in TSB supplemented with D-alanine, but no visible growth was detected in the absence of this compound. **C.** Relative expression levels of the *alr1* and *alr2* genes from the wild type strain and the isogenic mutants of *S. aureus* 132 analyzed by real-time RT-PCR. The relative expression level is calculated as $2^{-\Delta CT}$, where ΔCT is the difference between the crossing point target value and the crossing point reference value, normalized to the transcription levels of the housekeeping gene *gyrB*. The target is the strain indicated, whereas the reference is *S. aureus* 132 wild type in all cases. Data are the mean ± s.e.m.

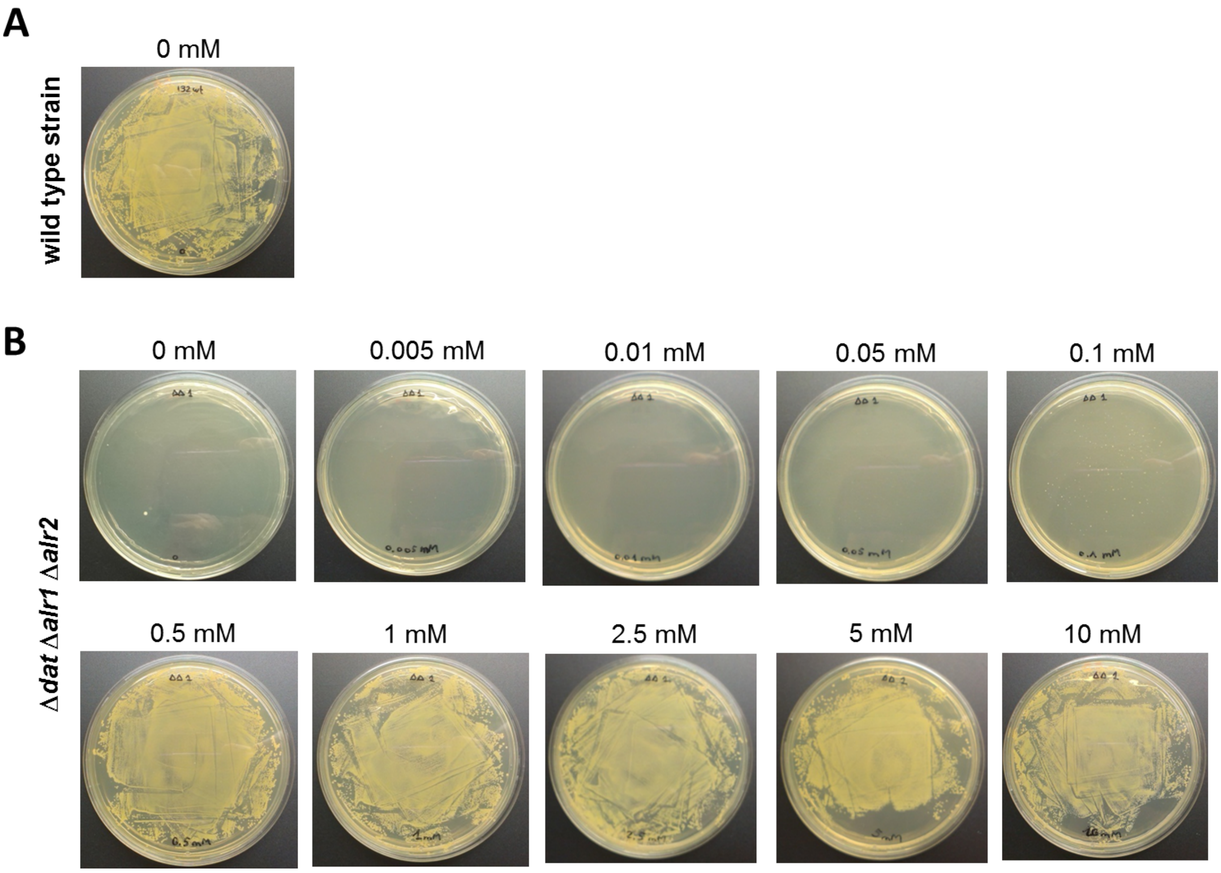

Figure S3. Determination of D-alanine concentration required for growing *S. aureus* 132 Δ*dat* Δ*alr1* Δ*alr2*. Log-phase cultures (OD_{600} = 0.2) of the wild type (**A**) and the triple mutant (**B**) strains were plated onto TSA without (0 mM) or with D-alanine at different concentrations (from 0.005 mM to 10 mM) as indicated.

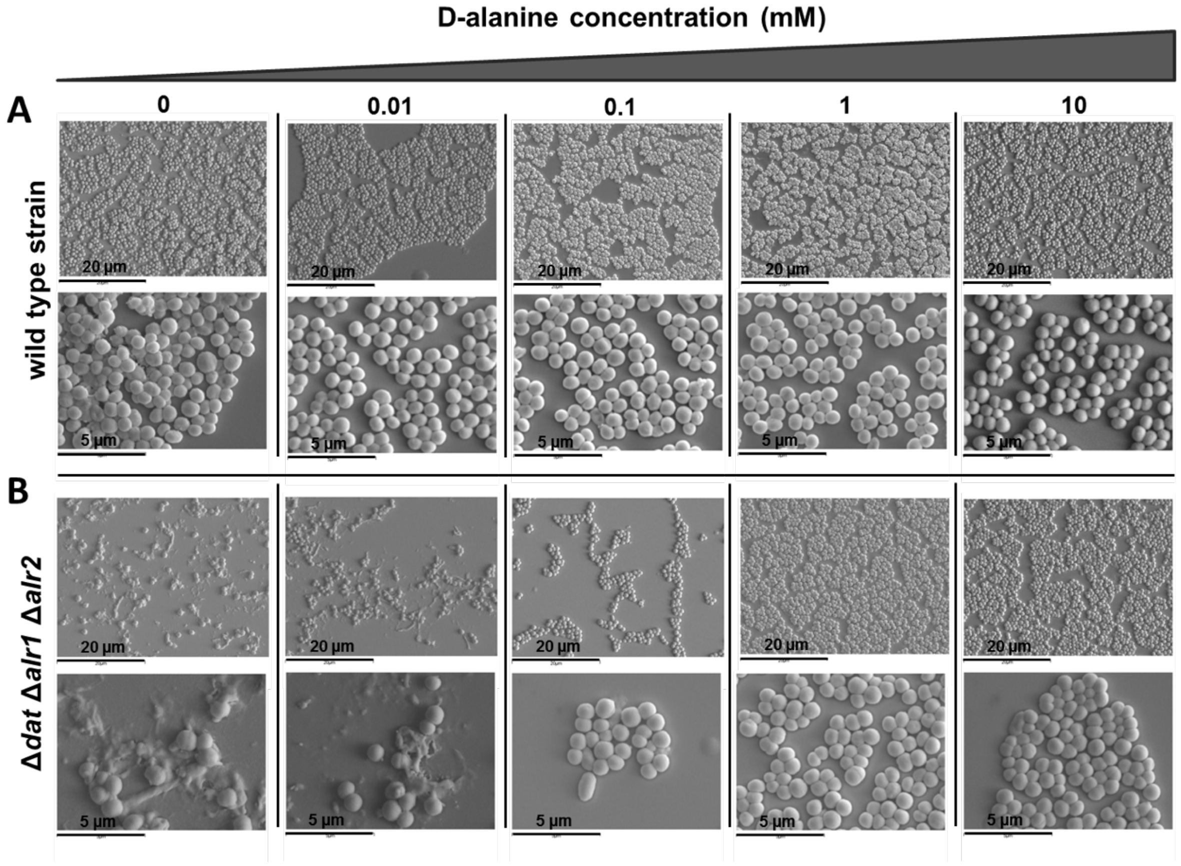

Figure S4. Morphological changes of *S. aureus* 132 Δ*dat* Δ*alr1* Δ*alr2* (B) with respect to the wild type strain (**A**) in the presence of different concentrations of D-alanine (from 0.01 to 10 mM). Images were taken with a scanning electron microscope. Bar indicates a scale of 5 µm or 20 µm.

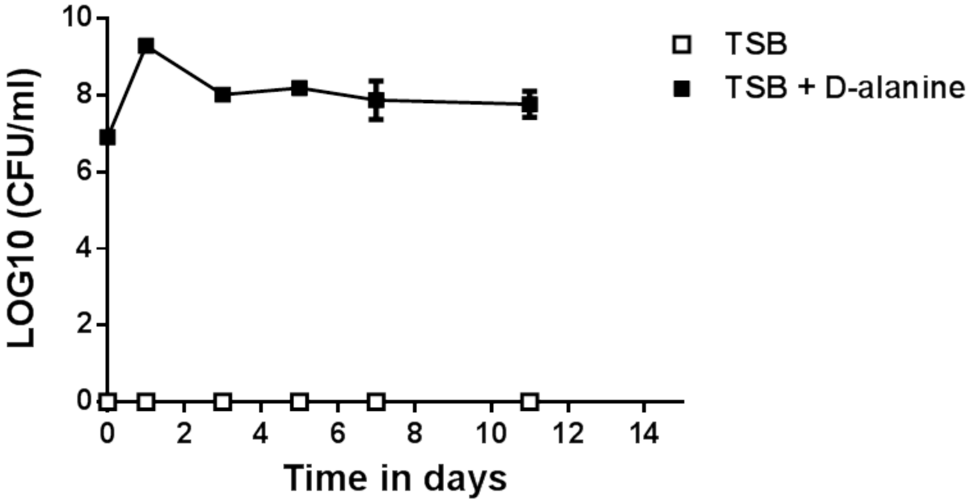

Figure S5. Evaluation of the phenotypic stability of *S. aureus* 132 Δ*dat* Δ*alr1* Δ*alr2* strain. Overnight cultures from the auxotroph mutant of *S. aureus* 132 were diluted at an initial OD_{600} of 0.02 into TSB supplemented with 5 mM D-alanine and incubated at 37°C under agitation (210 rpm) for up to 11 days. Aliquots were removed at different days, and the viable counts (Log_{10} CFU per milliliter) on TSA ( ) or TSA supplemented with 5 mM D-alanine (■) were determined. All experiments were performed in triplicate.

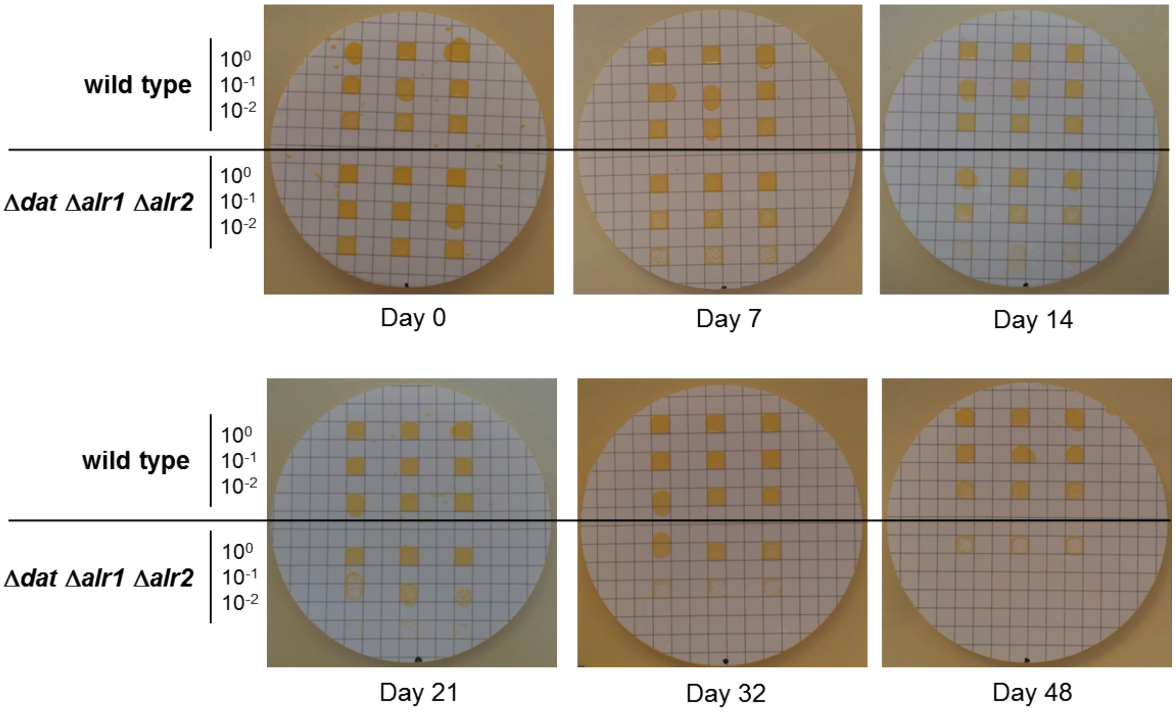

Figure S6. Survival of D-alanine auxotrophic strain exposed to dessication conditions. Cultures of the wild type 132 and 132 Δ*dat* Δ*alr1* Δ*alr2* strains at log-phase growth were washed and serially diluted in TSB. Drops of 5 μL were spotted on sterile membrane filters and maintained at room temperature. Thereafter, the filters were placed immediately (day 0) or after several days (7, 14, 21, 32, and 48 days) onto TSA plates containing 5 mM D-alanine and incubated at 37°C for 18 h. Cultures were performed in triplicate.
